# Gut ribotoxic stress responses facilitate dyslipidemia via metabolic reprogramming: an environmental health prediction

**DOI:** 10.7150/thno.88586

**Published:** 2024-01-20

**Authors:** Juil Kim, Hoyoung Jeong, Navin Ray, Ki-Hyung Kim, Yuseok Moon

**Affiliations:** 1 Laboratory of Mucosal Exposome and Biomodulation, Department of Integrative Biomedical Sciences, Pusan National University, Yangsan, Korea.; 2 Department of Obstetrics and Gynecology, College of Medicine, Pusan National University, Pusan National University, Busan, Korea.; 3 Biomedical Research Institute, Pusan National University, Busan, Korea.; 4 Graduate Program of Genomic Data Sciences, Pusan National University, Yangsan 50612, Korea.

**Keywords:** Ribotoxic stress responses, human inflammatory bowel diseases, dyslipidemia, LDL receptor, deoxynivalenol

## Abstract

**Rationale:** The gut and its accessory organ, the liver, are crucial determinants of metabolic homeostasis via the regulation of circulating lipids for cardiovascular health. In response to environmental insults, cells undergo diverse adaptation or pathophysiological processes via stress-responsive eukaryotic initiation factor 2 alpha (eIF2α) kinase signaling and subsequent cellular reprogramming. We noted that patients with inflammatory gut distress display enhanced levels of ribosomal stress-responsive eIF2α kinase, which is notably associated with lipid metabolic process genes. Based on an assumption that eukaryotic ribosomes are a promising stress-responsive module for molecular reprogramming, chemical ribosome-inactivating stressors (RIS) were assessed for their involvement in enterohepatic lipid regulation.

**Methods:** Experimental assessment was based on prediction using the clinical transcriptome and single-cell RNA-sequencing analysis of inflammatory bowel diseases and obesity. The prediction was verified using RIS exposure models of mice, gut organoids, and intestinal cells. The lipidomic profiling was performed to address RIS-induced intracellular fat alterations. Biochemical processes of the mechanisms were evaluated using RT-PCR, western blot analysis, luciferase reporter assays, and confocal microscopy of genetically ablated or chemically inhibited mice, organoids, and cells.

**Results:** Chemical RIS including deoxynivalenol promoted enterohepatic lipid sequestration while lowering blood LDL cholesterol in normal and diet-induced obese mice. Although ribosomal stress caused extensive alterations in cellular lipids and metabolic genes, the cholesterol import-associated pathway was notably modulated. In particular, ribosomal stress enhanced gut levels of the low-density lipoprotein receptor (*LDLR*) via both transcriptional and post-transcriptional regulation. Subsequently, LDLR facilitated enterohepatic cholesterol accumulation, leading to dyslipidemia in response to ribosomal stress. Moreover, genetic features of stress-responsive LDLR modulators were consistently proven in the inflammation- and obesity-associated gut model.

**Conclusion:** The elucidated ribosome-linked gut lipid regulation provides predictive insights into stress-responsive metabolic rewiring in chronic human diseases as an environmental health prediction.

## Introduction

In response to diverse environmental stressors, eukaryotic cells activate a common adaptive pathway, the integrated stress response (ISR), to restore cellular integrity or promote pathological processes. The core biochemical event in ISR is the phosphorylation of eukaryotic translation initiation factor 2 alpha (eIF2α) by the eIF2α kinase family, leading to the global translational arrest and induction of specific stress-responsive genes to achieve biological homeostasis 1, 2. Four stress-related mammalian protein kinases reportedly target the alpha subunit of eIF2: double-stranded RNA-dependent protein kinase R (PKR), RNA-dependent protein kinase-like ER kinase (PERK), eIF2α kinase general control non-repressed 2 (GCN2), and heme-regulated eIF2α kinase (HRI) 1, 2. In particular, eukaryotic ribosomes form a central platform of stress-responsive ISR regulation 3-5. Of note, viral infection-induced double-stranded RNA or xenobiotic-induced 28S rRNA damage facilitates ribosomal binding to dsRNA-binding domains of EIF2AK2 and induces enzymatic activation 5-7. In response to ribosomal stress, the ribosomal 40S subunit serves as a scaffold for EIF2AK2 action, which facilitates the expression of stress adaptive genes including pro-inflammatory genes via subsequent signaling activation of kinases such as mitogen-activated protein kinases (MAPKs) 7-9.

The human intestines play a key role in maintaining lipid homeostasis since it is the only site for the absorption of dietary sterols as the single most active location for cholesterogenesis 10, 11. Moreover, the small intestine is the second most crucial organ for the uptake and degradation of circulating low-density lipoprotein (LDL), contributing to lowering plasma cholesterol levels 12, 13. Additionally, messengers from the small intestine can control hepatic metabolic processes to adequately address large fluctuations in dietary cholesterol intake and hypercholesterolemia that can progress to cardiovascular diseases 14, 15. However, persistent dyslipidemia is strongly associated with a high incidence of human inflammatory bowel diseases, still being of unknown etiology. Gastrointestinal dysregulation of lipid metabolism has been documented in patients with ulcerative gut injuries 16, 17. Compared with healthy individuals, patients with inflammatory bowel disease (IBD), including Crohn's disease (CD) and ulcerative colitis (UC), tend to experience dyslipidemia and diverse metabolic disorders, such as the dysregulation of circulating cholesterol. Notably, persistent low levels of serum LDL cholesterol are strongly associated with a high incidence of CD, still being of unknown etiology 18, 19. Considering the frontline defense against environmental and dietary insults, the metabolic profiling of the alimentary tract needs to be extensively evaluated.

Environmental stress-induced 28S rRNA damage and subsequent stress-sentinel pathways are potent etiological factors involved in inflammatory or malignant distress in the experimental models 20-27. Humans are frequently exposed to ribosome-damaging environmental insults including microbe- and plant-derived xenobiotics, ultraviolet light, certain tumor promoters, and antibiotics 28. In the present study, ISR-linked stress was examined in alimentary tract-associated organs such as the gut and liver, both major sites of metabolic homeostasis in response to dietary and environmental factors. In particular, the ribotoxic stress responses were associated with gastrointestinal lipid regulation to simulate the clinical metabolic distress since the ribosome is a potent sentinel organelle of ISR as mentioned. This study determined whether ISR affects the gastrointestinal lipid profile and is involved in the regulation of pathophysiological outcomes. ISR-associated gut fat modulation would provide new insights into stress-responsive metabolic alteration in chronic human diseases.

## Results

### Gut ribosomal dysfunction is associated with fat deposition and dyslipidemia

**Figure 1:**
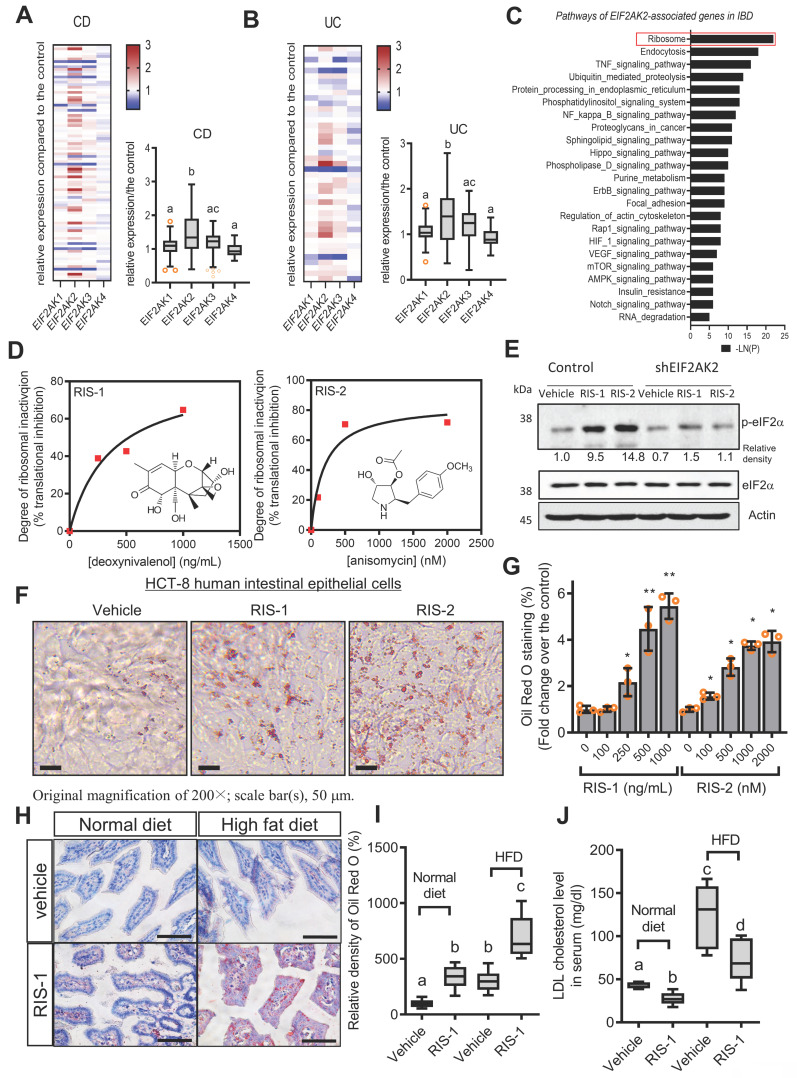
** Association of ribosome-linked stress responses with gut distress.** (A-B) Intestinal levels of eIF2α kinase genes were compared in patients with different types of IBD (Crohn's disease [CD, A] and ulcerative colitis [UC, B]) (gse117993 (*Denson*'s, n = 190)), and the quantitative analysis is presented in each right graph. Results are shown as box-and-whisker plots (Tukey), and different letters over each box represent significant differences between groups (*p* < 0.05 using one-way ANOVA with the Newman-Keuls post hoc test). (C) KEGG-based functional annotations of EIF2AK2-related pathways in patients with IBD (GEO ID: gse117993, n = 190). (D) HCT-8 cells were exposed to serial concentrations of RIS-1 or RIS-2 for 24 h and changes in total protein concentration per cell (protein synthesis) were measured. Levels of total protein synthesis for 24 h in the absence or presence of the specific ribosome-directed inactivating chemical (RIS-1 or RIS-2) were compared. (E) The control vector- or EIF2AK2 shRNA expression plasmid (shEIF2AK2)-transfected HCT-8 cells were treated with the vehicle, 1000 ng/mL RIS-1, or 2 μM RIS-2 for 5 min. The cell lysates were subjected to western blot analysis. (F-G) HCT-8 cells were treated with the vehicle, 1000 ng/mL RIS-1, or 2 μM RIS-2 for 24 h. Intracellular lipid droplets were stained with Oil Red O and quantified using the ImageJ software. The graph indicates dose responses of the lipid droplet levels, and the asterisks represent a significant difference from each vehicle control (G, *p < 0.05 and **p < 0.01). The microscopic analysis was performed at the original magnification of 200×; scale bar(s), 50 μm. (H-J) Three-week-old C57 BL6J mice were fed normal chow or 60% high-fat diet (HFD) from weaning for 12 weeks. Mice were then treated with the vehicle or 25 mg/kg RIS-1 (25 mg/kg) for 24 h via oral gavage. Representative Oil Red O staining of the small intestine at the original magnification of 400×; scale bar(s), 50 μm (H). Relative quantitative values of Oil Red O-positive lipid droplets were quantified using ImageJ software (I). Serum LDL cholesterol was measured (J). Results are shown as box-and-whisker plots (Tukey), and different letters over each box represent significant differences between groups (*p* < 0.05 using one-way ANOVA with the Newman-Keuls post hoc test). LDL, low-density lipoprotein. eIF2α, eukaryotic initiation factor 2 alpha.

Based on the assumption that ISR-linked cellular processes are involved in modulating gastrointestinal distress, four global stress-related mammalian eIF2α kinases, including *HRI* (*EIF2AK1*), *PKR* (*EIF2AK2*), *PERK* (*EIF2AK3*), and *GCN2* (*EIF2AK4*), were evaluated using a clinical transcriptome dataset (Figure 1A-1B). Among these, *EIF2AK2* level was notably elevated in patients with UC and CD when compared with levels of other mammalian eIF2α kinases. Moreover, gene-based functional prediction of the disease mechanism demonstrated the involvement of EIF2AK2-associated gene clusters in the regulation of the ribosomal process in patients with ulcerative gut distress (Figure 1C). Herein, EIF2AK2-linked stress responses were simulated in cell- and animal-based experimental models of ribosomal stress 23-25, given that stress-driven ribosomal stalling can trigger eIF2α-mediated global translational inhibition via EIF2AK2. Based on the intestinal epithelial cell culture conditions, the extent of xenobiotic-induced ribosomal inactivation was titrated to the magnitude of translational inhibition using specific ribosome-inactivating stressors (RIS) such as deoxynivalenol (RIS-1) or anisomycin (RIS-2) (Figure 1D). In the following evaluations, we treated cells with each RIS at a dose required to inactivate approximately 70% of the intrinsic ribosomal function (protein translation). Moreover, the chemical-induced ribosomal dysfunction triggered eIF2α phosphorylation, which was confirmed dependent on EIF2AK2 in HCT-8 cells (Figure 1E). HCT-8 cells have become a widely adopted model for studying inflammatory and infectious diseases in human intestinal epithelia 29, 30. In particular, the HCT-8 cell line was derived from the ileocecum region that exhibits high vulnerability to ribosome-inactivating stress 31, 32.

The ribosome-linked stress responses were further assessed in association with gut lipid regulation. To determine changes in cellular lipid, ribosome-inactivated intestinal epithelial cells were stained with Oil Red O. Chemical-induced ribosomal inactivation dose-dependently enhanced intracellular lipid deposition in HCT-8 human intestinal epithelial cells (Figure 1F-1G). Cells were treated with RIS to simulate the effects of EIF2AK2-triggering ribosomal stress. Furthermore, HepG2 hepatocytes originating from the human liver, another cholesterol-regulating organ, exhibited dose-dependent lipid accumulation in response to chemical ribosome inactivation (Figure S1A-S1B). In addition to its effect on the normal state, ribosomal stress was evaluated in diet-induced obese animals. Gut exposure to RIS enhanced intestinal lipid deposition in diet-induced obese animals (Figure 1H-1I). Ribosomal stress-induced fat accumulation was also observed in the liver (Figure S1C-S1D). In contrast to fat accumulation in the gut and liver, the ribosomal stress significantly reduced serum cholesterol levels in mice (Figure 1J). Although ribosomal stress caused a notable reduction in blood cholesterol, it was not associated with the body weight of the mice (Figure S1E). Moreover, there were marginal changes in the histopathological scores in inflammation, crypt loss, and ulceration despite slight reductions in crypt length in the small intestine in response to the ribosomal stress (Figure S2A-S2E). However, as implicated in the reduction of blood cholesterol, ribosomal stress significantly enhanced enterohepatic tissue cholesterol in both normal and diet-induced obese mice, indicating that enhanced tissue cholesterol deposition led to plasma cholesterol lowering (Figure S3A-S3D).

### Ribosomal stress alters the intracellular lipid metabolic processes

**Figure 2:**
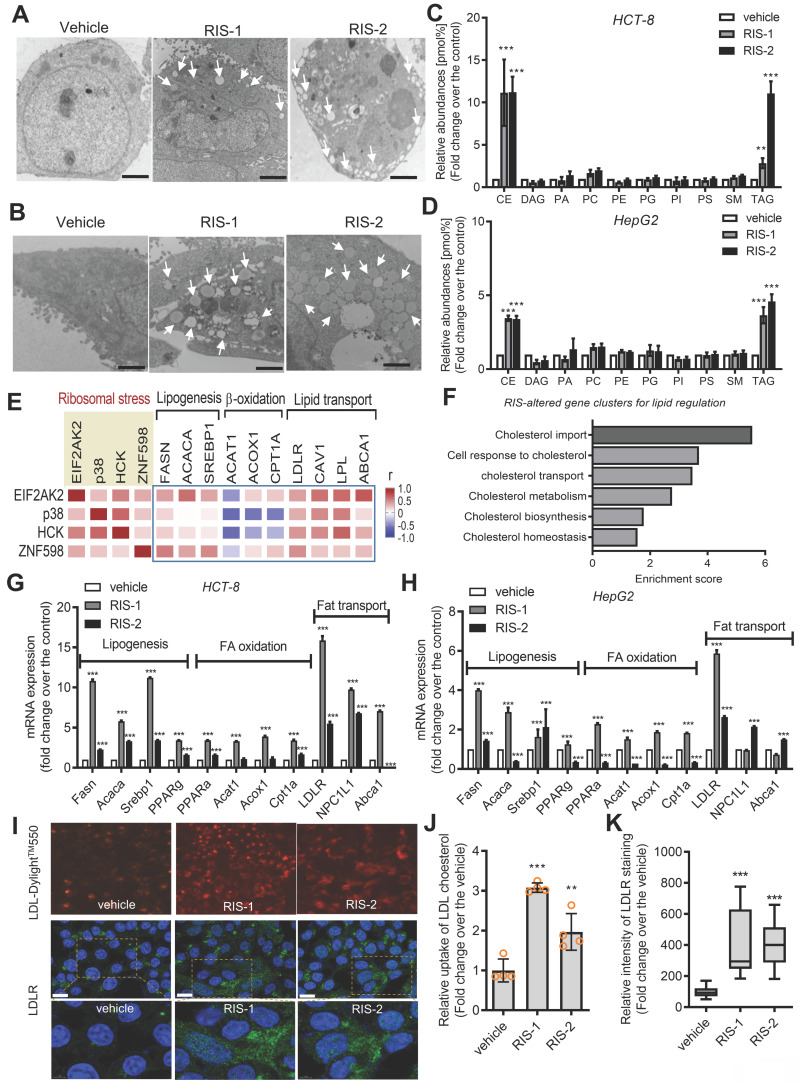
** Effects of ribosomal inactivation on intracellular fat and gene profiles.** (A-D) HCT-8 (A and B) and HepG2 (C and D) cells were treated with the vehicle, 1000 ng/mL RIS-1, or 2 mM RIS-2 for 24 h. (A and B) Lipid droplets were detected using a transmission electron microscope (TEM) at the original magnification x1000 (scale bar(s), 2 mm) with white arrows indicating lipid droplets in each cell. (C and D) Total lipids from the whole cell lysate were analyzed for lipidomic profiling using mass spectrometry (performed by Lipotype GmbH, Dresden, Germany). (E) *Pearson*'s correlation analysis of relationships between transcription levels of key components of the ribotoxic stress responses and lipid metabolism in patients with IBD (gse117993 (*Denson*'s, n = 190)). Correlation matrix visualization was generated using the corrplot function of the R-package (R Foundation for Statistical Computing, Vienna, Austria. URL: https://www.R-project.org/). Correlations of transcriptional expression among genes were interpreted according to a general guideline for Pearson's coefficient value: r > 0.7, high (+); 0.5 < r < 0.7, moderate (+); 0.3 < r < 0.5, moderate (+) or low (+); 0.1 < r < 0.3, low (+); -0.1 < r < 0.1, negligible; -0.3 < r < -0.1, low (-). (F) HCT-8 cells were treated with the vehicle or 500 ng/mL RIS-1 for 1 h. Functional gene ontology using RNA sequencing analysis of the cellular mRNA was evaluated based on *p* values for each category. (G-H) HCT-8 (G) and HepG2 (H) cells were treated with the vehicle, 1000 ng/mL RIS-1, or 2 μM RIS-2 for 24 h. Each mRNA was measured using reverse transcription-quantitative PCR. (I-K) LDL uptake into human intestinal epithelial cells (upper panels) and cellular LDLR expression (lower pannels) were measured using LDL-Dylight^TM^ 550 (red) and DyLight^TM^-488-conjugated anti-LDLR antibody (green), respectively. The microscopic analysis was performed at the original magnification of 200× (scale bar(s), 50 μm) with each quantitative graph (J and K, *p < 0.05, **p < 0.01, and ***p < 0.001). LDL, low-density lipoprotein; LDLR, low-density lipoprotein receptor; CE, cholesterol esters; DAG, diacylglycerol; PA, phosphatidylcholine; PC, phosphatidylcholine; PE, phosphatidylethanolamine (-ether); PG, phosphatidylglycerol; PI, phosphatidylinositol; PS, phosphatidylserine; SM, sphingomyelin; TAG, triacylglycerol.

Intracellular lipid accumulation was closely observed using transmission electron microscopy (TEM). Ribosomal inactivation increased the number of lipid droplets in human intestinal epithelial cells and hepatocytes (Figure 2A and 2B, respectively). We further analyzed the intracellular lipid composition of ribosome-insulted intestinal and liver cells by performing lipidomic analysis. In particular, ribosomal insult markedly elevated cholesteryl ester and triacylglycerol levels, although some were found to be downregulated or marginally altered (Figure 2C and 2D). Therefore, on an assumption that cellular ribosomes are a promising stress-responsive module for lipid regulation, *EIF2AK2* and the ribotoxic stress response-related genes were further compared with components of lipid metabolic processes including lipogenesis, fatty acid oxidation, and lipid transport (Figure 2E). Of note, the ribosomal stress-featured genes exhibited a positive correlation with lipid transport-linked genes despite some negative correlations with β-oxidation genes such as acetyl-CoA acetyltransferase 1 (*ACAT1*) in patients with IBD. Considering these associations, lipid regulation was further examined in the stress-insulted gut cells. Despite the global translational arrest, the functional pattern analysis of mRNA expression revealed that genes involved in cholesterol import-related responses were dramatically altered in ribosome-insulted human intestinal epithelial cells (Figure 2F). Although ribosomal stress altered genes involved in lipogenesis and fatty acid oxidation, the cholesterol-rich LDLR for cholesterol uptake was notably induced in intestinal and hepatic epithelial cells (Figure 2G and 2H, respectively). To account for intracellular cholesterol accumulation, we assessed LDL cholesterol uptake via LDLR in human intestinal epithelial cells. Although the complete culture media contained cholesterol-rich LDL particles 33, we added fluorescent dye-labeled LDL to the media to monitor its translocation into human cells. Notably, ribosomal insult enhanced the intestinal epithelial uptake of labeled exogenous LDL and upregulated LDLR expression (Figure 2I-2K). LDLR-mediated uptake of LDL-cholesterol accounts for notable increases in intracellular conversion to cholesteryl ester and triacylglycerol in ribosome-insulted cells.

### Gut ribosomal stress differentially enhances LDLR levels in the gut cell population

**Figure 3:**
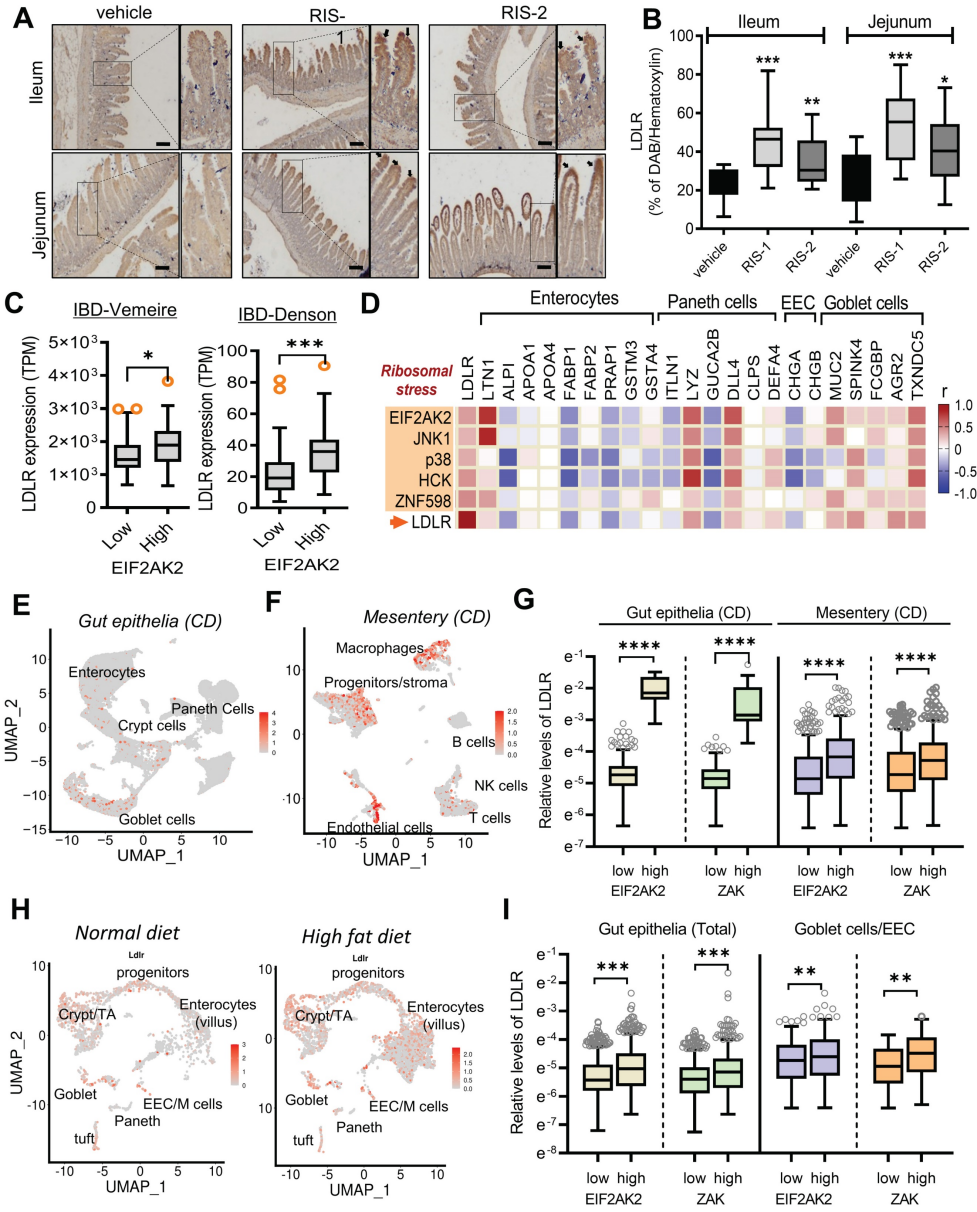
** The ribotoxic stress responses modulate intestinal LDLR expression.** (A-B) Mice were treated with the vehicle or 25 mg/kg RIS-1 for 24 h via oral gavage. Ileal and jejunal tissue specimens were subjected to immunohistochemistry to detect LDLR expression (A). The microscopic analysis was performed at the original magnification of 100× (scale bar(s), 100 μm), followed by quantitative analysis using HistoQuest software (B). (C) *LDLR* expression was assessed in patients with IBD (gse75214 (*Vemeire*'s, n = 194) and gse117993 (*Denson*'s, n = 190)). Based on *EIF2AK2* levels, we selected samples exhibiting the 50 highest and 50 lowest levels, which were further compared for *LDLR* levels. Results are shown as a box-and-whisker plot (Tukey), and asterisks (*) indicate significant differences from the low expression group (*p < 0.05, ** *p <* 0.01, *** *p <* 0.001). (D) *Pearson*'s correlation analysis of relationships between transcription levels of critical components of the ribotoxic stress responses and cell-type-specific markers in patients with IBD (gse117993 (*Denson*'s, n = 190)). Correlation matrix visualization was generated using the corrplot function of the R-package (R Foundation for Statistical Computing, Vienna, Austria. URL: https://www.R-project.org/). Correlations of transcriptional expression among genes were interpreted according to a general guideline for Pearson's coefficient value: r > 0.7, high (+); 0.5 < r < 0.7, moderate (+); 0.3 < r < 0.5, moderate (+) or low (+); 0.1 < r < 0.3, low (+); -0.1 < r < 0.1, negligible; -0.3 < r < -0.1, low (-). IBD, inflammatory bowel disease; LDL, low-density lipoprotein; LDLR, low-density lipoprotein receptor. (E-G) Analysis based on scRNA-Seq dataset from patients with CD (gse202052 (E) and gse156776 (F)). (E-F) UMAP clusters for gse202052 (E) and gse156776 (F). (G) Based on *EIF2AK2* or* ZAK* levels, we selected samples exhibiting the 500-1500 highest and 500-1500 lowest levels, which were further compared for *LDLR* levels. Results are shown as a box-and-whisker plot (Tukey), and asterisks (*) indicate significant differences from the low expression group (**** *p <* 0.0001). (H-I) Analysis based on scRNA-Seq dataset from mouse intestine fed with HFD for 7 days (gse199776). (H) UMAP clusters. (I) Based on *EIF2AK2* or* ZAK* levels, we selected samples exhibiting the 500 highest and 500 lowest levels, which were further compared for *LDLR* levels. Results are shown as a box-and-whisker plot (Tukey), and asterisks (*) indicate significant differences from the low expression group (** *p <* 0.01, *** *p <* 0.001).

LDLR expression in human intestinal epithelial cells was confirmed in ribosome-inactivated mice. The ribosome-insulted small intestines (ileum and jejunum) exhibited enhanced LDLR expression, particularly in the epithelial lining (Figure 3A and 3B). Following an in-depth examination of immunohistochemical staining, we found that LDLR expression was notably enhanced in the upper villus of epithelia, corresponding to highly differentiated enterocytes and goblet cells, rather than in the lower and crypt sections containing proliferating or progenitor cells. Furthermore, we quantified the effects of different doses of ribosome inactivators on LDLR expression in human intestinal epithelial cells and hepatocytes. Quantitative analysis of LDLR mRNA expression revealed that both RIS-1 and RIS-2 dose-dependently increased LDLR expression (Figure S4A-S4B).

Based on the assumption that EIF2AK2-activating ribosomal stress plays a crucial role in cholesterol regulation in the gastrointestinal tract, LDLR expression was assessed in patients with IBD. Patients with UC and CD exhibited higher LDLR levels than controls (Figure S4C-S4D). Notably, patients with UC tended to present higher LDLR expression than those with colon-only CD (cCD) and ileocolonic CD (iCD). Moreover, subjects with high levels of EIF2AK2 displayed enhanced LDLR expression (Figure 3C). We further assessed expression patterns of LDLR and ribotoxic stress response-linked genes in association with cell-type-specific markers in the gut (Figure 3D). EIF2AK2-associated ribotoxic stress response genes displayed a positive correlation with *LDLR* levels. Moreover, subjects with high levels of goblet cell markers tended to present increased expressions of *EIF2AK2*, ribosomal stress markers, and *LDLR* in human IBD. Single-cell sequencing-based evaluation of gut tissues in patients with CD verified notable expressions of *LDLR* in the goblet cells as well as enterocytes (Figure 3E). Moreover, chronic inflammation-induced epithelial barrier disruption leads to mucosa-associated bacterial translocation to the mesentery and expansion of inflammatory cells and creeping fat in the mesenteric adipose tissue (MAT) 34. In addition to the gut epithelia, MAT displayed enhanced levels of LDLR notably in the macrophages and progenitor cells in patients with CD (Figure 3F and S5A-S5C). Moreover, we predicted associations of the ribotoxic stress responses with LDLR expression. Subjects with high expression of EIF2AK2 and ZAK, ribosomal stress-responsive markers, exhibited increased levels of LDLR in both gut epithelia and MAT (Figure 3G).

In addition to the gut inflammatory diseases, we further evaluated LDLR expression in the gut of diet-induced obese mice as a metabolically insulted model. Single-cell sequencing-based evaluation of gut tissues demonstrated that the high-fat diet (HFD) feeding caused prevalent increases in LDLR levels in different types of intestinal cells at a glance (Figure 3H). HFD feeding enhanced whole intestinal levels of LDLR in a time-dependent manner in the gut (Figure S5D). A quantitative comparison of cell type-specific expression suggested that HFD-linked LDLR levels were relatively higher in goblet cells and enteroendocrine cells (EEC) than those in crypt cells including progenitor cells and Paneth cells (Figure S5E). Moreover, we predicted associations of the ribotoxic stress responses with LDLR expression in the obesity model. Subjects with high expression of ribosomal stress-responsive markers (EIF2AK2 and ZAK) showed increased levels of LDLR in the gut epithelia (Figure 3I). Taken all, the ISR-linked ribosomal stress was positively correlated with LDLR expression in the gut and mesentery with inflammatory or metabolic distress, particularly in a cell-type-linked manner.

### Gut ribosomal stress-induced fat sequestration via modulation of LDLR

**Figure 4:**
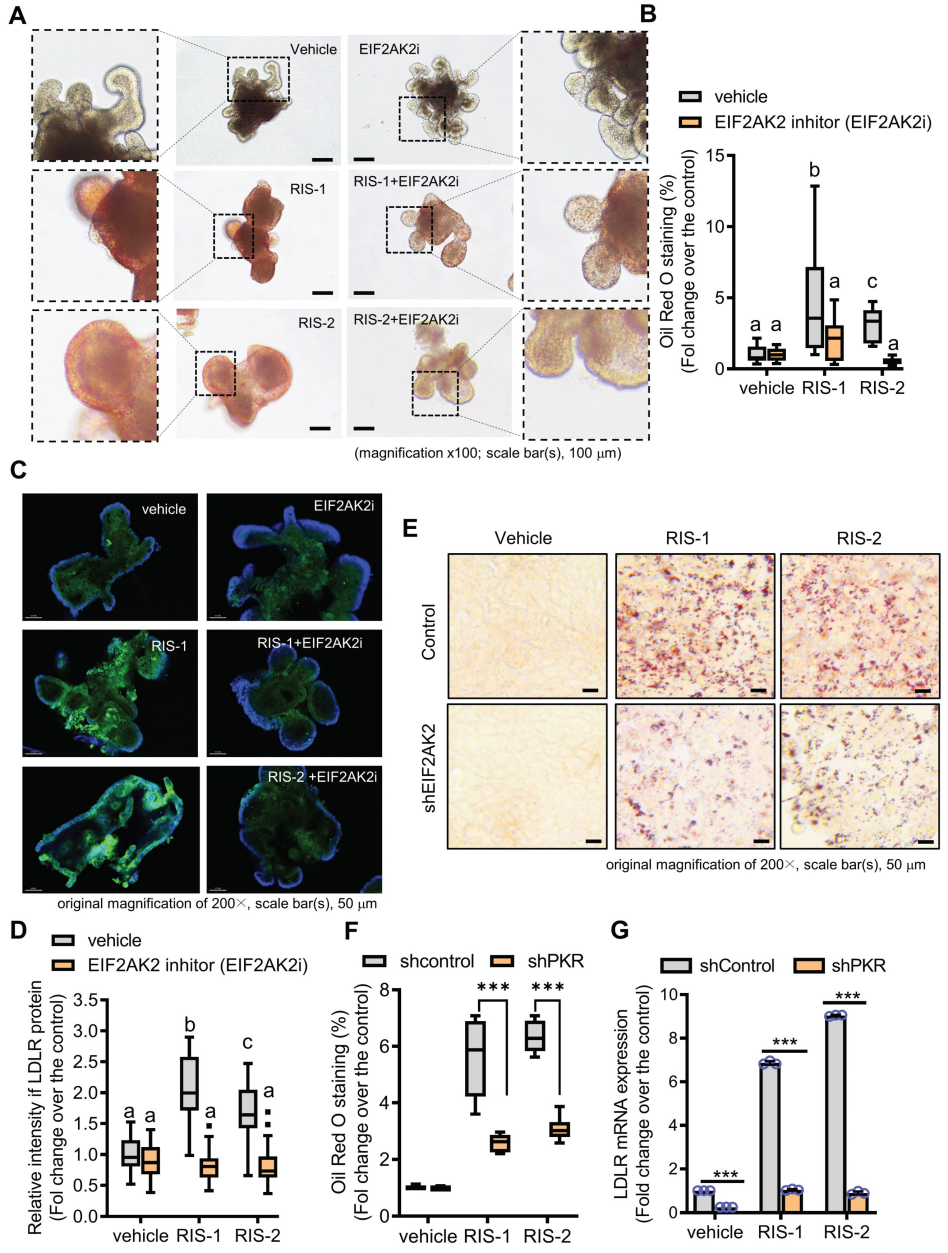
** EIF2AK2 mediates LDLR expression in the ribosome-insulted gut.** (A-D) Small intestine-derived organoids from the wild-type mice were treated with vehicle, RIS-1 (500 ng/mL), RIS-2 (1 μM), or EIF2AK2 inhibitor (0.5 μM, EIF2AK2i) for 24 h. (A) Representative Oil Red O staining for the intestinal organoids. The microscopy analysis was performed at the original magnification of 100×; scale bar(s), 100 μm. (B) The graph shows relative quantitative values of Oil Red O-positive lipid droplets in the gut organoids using ImageJ software. Different letters over each bar represent significant differences between groups (*p* < 0.05). (C) The confocal microscopic analysis of LDLR expression was performed at the original magnification of 200×; scale bar(s), 50 μm. (D) The graph shows the relative quantitative values for LDLR protein in the intestinal organoids using ImageJ software. Different letters over each bar represent significant differences between groups (*p* < 0.05). (E-G) HCT-8 cells transfected with the negative control vector or shEIF2AK2 were treated with the vehicle, 1000 ng/mL RIS-1, or 2 μM RIS-2. (E) Intracellular lipid droplets were stained with Oil Red O and visualized using a light microscope. The microscopy analysis was performed at the original magnification of 200×; scale bar(s), 50 μm. (F) The graph shows relative quantitative values of Oil Red O-positive lipid droplets in cells using ImageJ software (***p < 0.001). (G) HCT-8 cells expressing the negative control or EGR1-specific shRNA were treated with vehicle, 1000 ng/mL RIS-1, or 2 μM RIS-2 for 2 h. LDLR mRNA was measured using reverse transcription-quantitative PCR (***p < 0.001).

We further assessed whether the EIF2AK2-activating signal modulates the gut LDLR expression in the ribosome-insulted gut organoid and cell models. Chemical-induced ribosomal stress enhanced fat accumulation in the mouse intestinal organoids, which was attenuated by EIF2AK2 inhibition using a specific chemical inhibitor (EIF2AK2i) (Figure 4A-4B). Moreover, Ribosomal stress-induced LDLR expression was notably dependent on EIF2AK2 signaling in the gut organoids (Figure 4C-4D). Genetic ablation of EIF2AK2 by its shRNA expression also attenuated fat deposition (Figure 4E-4F) and LDLR expression (Figure 4G) in the ribosome-insulted human intestinal cells, verifying a positive regulation of LDLR by EIF2AK2 signaling in response to the ribosomal dysfunction.

**Figure 5:**
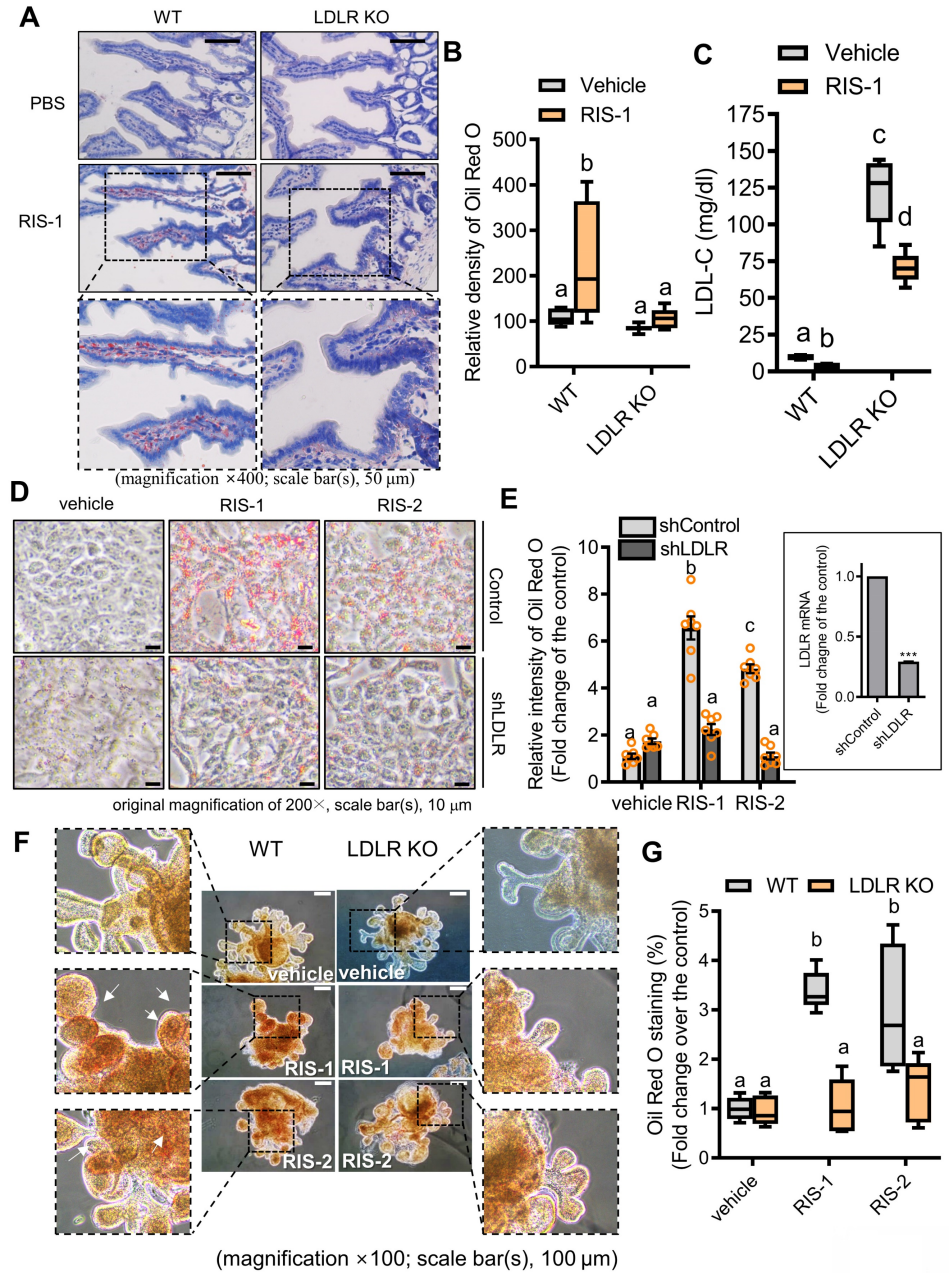
** LDLR mediates fat deposition in the gut** (A) Ten-week-old male C57BL/6J wild-type or LDLR knockout (KO) mice were treated with the vehicle or 25 mg/kg RIS-1 for 24 h via oral gavage. Representative Oil Red O staining for the small intestine. (B) Relative quantitative values of Oil Red O-positive lipid droplets in small intestines using ImageJ software. The microscopy analysis was performed at the original magnification of 400×; scale bar(s), 50 μm. (C) Measurement of serum LDL cholesterol. Results are shown as box-and-whisker plots (Tukey), and different letters over each box represent significant differences between groups (*p* < 0.05 using one-way ANOVA with the Newman-Keuls post hoc test). (D-E) HCT-8 cells transfected with the negative control vector or shLDLR were treated with the vehicle, 1000 ng/mL RIS-1, or 2 μM RIS-2. Intracellular lipid droplets were stained with Oil Red O and visualized using a light microscope (D). The microscopic analysis was performed at the original magnification of 200×; scale bar(s), 10 μm (left panel). (E) The graph shows relative quantitative values of Oil Red O-positive lipid droplets in cells using ImageJ software (E). The right boxed graph represents the suppression of LDLR mRNA expression by shLDLR (***p < 0.001). (F) Small intestine-derived organoids from wild-type or LDLR-KO mice were treated with vehicle, RIS-1 (500 ng/mL), or RIS-2 (1 μM) for 24 h. Representative Oil Red O staining for small intestine organoids. The microscopy analysis was performed at the original magnification of 100×; scale bar(s), 100 μm. Different letters over each bar represent significant differences between groups (*p* < 0.05). (G) Relative quantitative values of Oil Red O-positive lipid droplets in organoids using ImageJ software. Results are shown as box-and-whisker plots (Tukey), and different letters over each box represent significant differences between groups (*p* < 0.05 using one-way ANOVA with the Newman-Keuls post hoc test). LDL, low-density lipoprotein; LDLR, low-density lipoprotein receptor.

To further confirm our hypothesis that lipid accumulation induced by ribosome inactivation depends on LDLR-mediated cholesterol uptake, we assessed the effects of LDLR deficiency on lipid deposition in ribosome-insulted intestinal epithelial cells and hepatocytes. LDLR knockout (LDLR-KO) mice showed markedly attenuated lipid accumulation in the ribosome-insulted gut and liver tissues (Figure 5A-5B and S6A-S6B). As LDLR recognizes apoproteins embedded in the outer layer of LDL particles, LDLR does not mediate the uptake of diet-derived free cholesterol or bile-derived cholesterol from the gastrointestinal lumen 35. Therefore, we focused on the LDLR-mediated enterohepatic regulation of cholesterol from the circulation or tissues rather than from the gut lumen. Whereas LDLR deficiency attenuated lipid deposition in tissues, serum LDL cholesterol levels were markedly elevated in LDLR-KO mice (Figure 5C), indicating LDLR-mediated tissue cholesterol deposition in the ribosome-insulted gut and liver. Consistent with the *in vivo* evaluation, LDLR suppression using respective small hairpin RNA (shRNA) showed reduced lipid deposition in ribosome-insulted intestinal epithelial cells and hepatocytes (Figure 5D-5E and S6C-S6D). Moreover, the gut organoid-based model verified LDLR-dependent lipid accumulation (Figure 5F and 5G). Taken together, ribosome inactivation could enhance cholesterol uptake and its subsequent deposition by upregulating LDLR expression in gut and liver cells.

### Ribosomal stress-responsive early growth response 1 (EGR1) and sterol regulatory element (SRE)-binding protein 2 (SREBP2) are involved in LDLR induction and subsequent fat deposition

**Figure 6:**
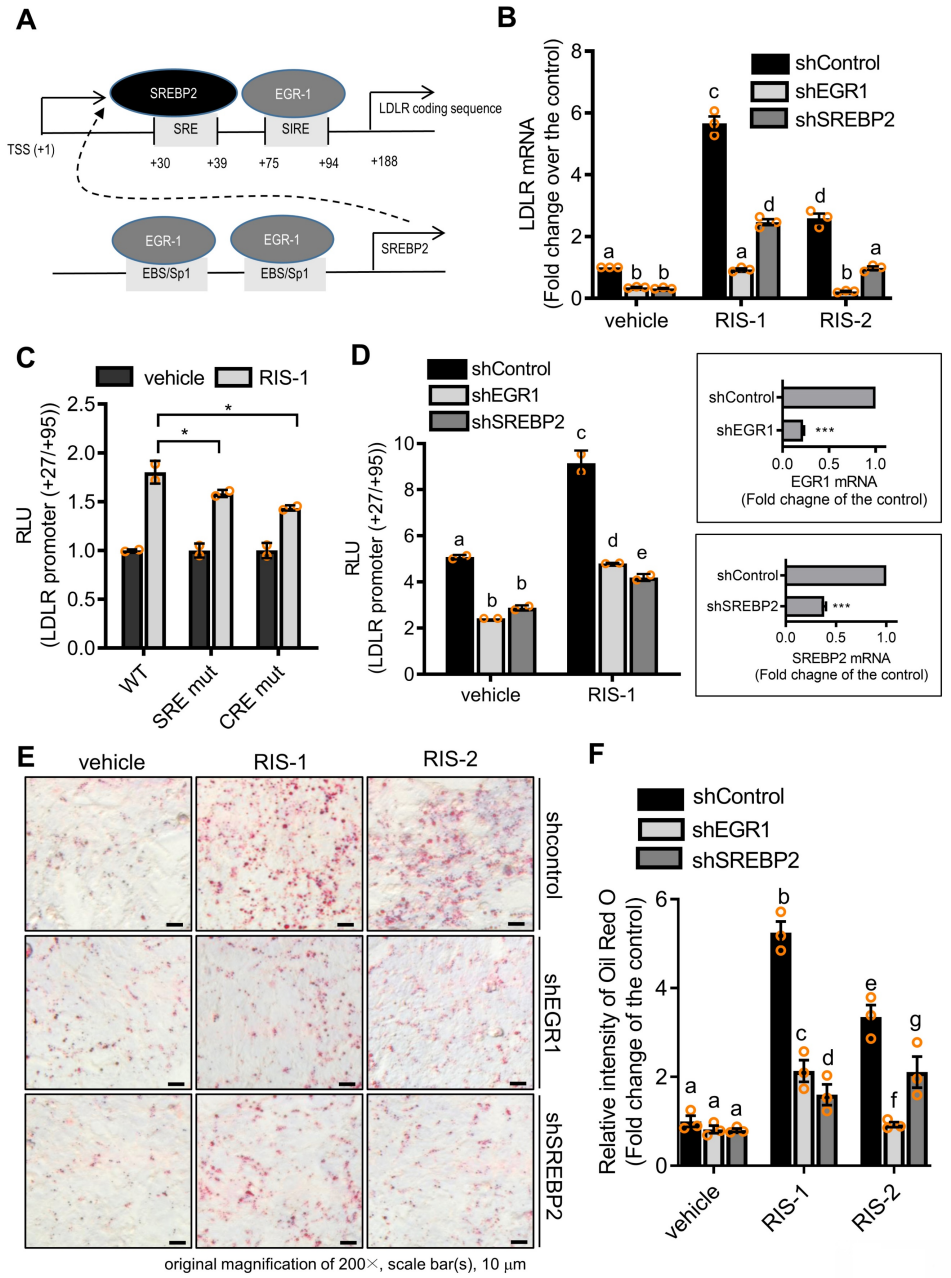
** Transcriptional regulation of LDLR expression in response to ribosomal inactivation.** (A) A putative scheme for ribosomal stress-induced transcriptional activation of LDLR expression via SREBP2 activation and EGR1 induction. (B) HCT-8 cells expressing the negative control vector, EGR1-, or SREBP2-specific shRNA were treated with vehicle, 1000 ng/mL RIS-1, or 2 μM RIS-2 for 2 h. LDLR mRNA was measured using reverse transcription-quantitative PCR. Figures in the box represent the inhibition of EGR-1 and SREBP2 mRNA by each shRNA (***p < 0.001). (C) HCT-8 cells transfected with the wild-type (wt) or the mutants (SRE mt and CRE mt) of LDLR promoter-containing plasmids were treated with vehicle or 1000 ng/mL RIS-1 for 6 h, and the cellular luciferase activity was measured (*p < 0.05). (D) EGR1- or SREBP2-deficient cells were transiently transfected with wt LDLR promoter-containing plasmid and then treated with vehicle or 1000 ng/mL RIS-1 for 6 h to measure the cellular LDLR promoter activity. Different letters over each bar represent significant differences between the two groups (*p* < 0.05). The right boxed graphs show suppression of mRNA expression by each shRNA (***p < 0.001). (E-F) HCT-8 cells expressing the negative control vector, EGR1-, or SREBP2-specific shRNA were treated with vehicle, 1000 ng/mL RIS-1, or 2 μM RIS-2 for 2 h. Intracellular lipid droplets were stained with Oil Red O and visualized using a light microscope at the original magnification of 200×; scale bar(s), 50 μm (E). The right graph shows the relative quantitative values of Oil Red O-positive lipid droplets, and different letters over each bar represent significant differences between the two groups (F, *p* < 0.05 using one-way ANOVA with the Newman-Keuls post hoc test). EGR1, early growth response 1; LDLR, low-density lipoprotein receptor; SREBP2, sterol regulatory element-binding protein 2.

**Figure 7:**
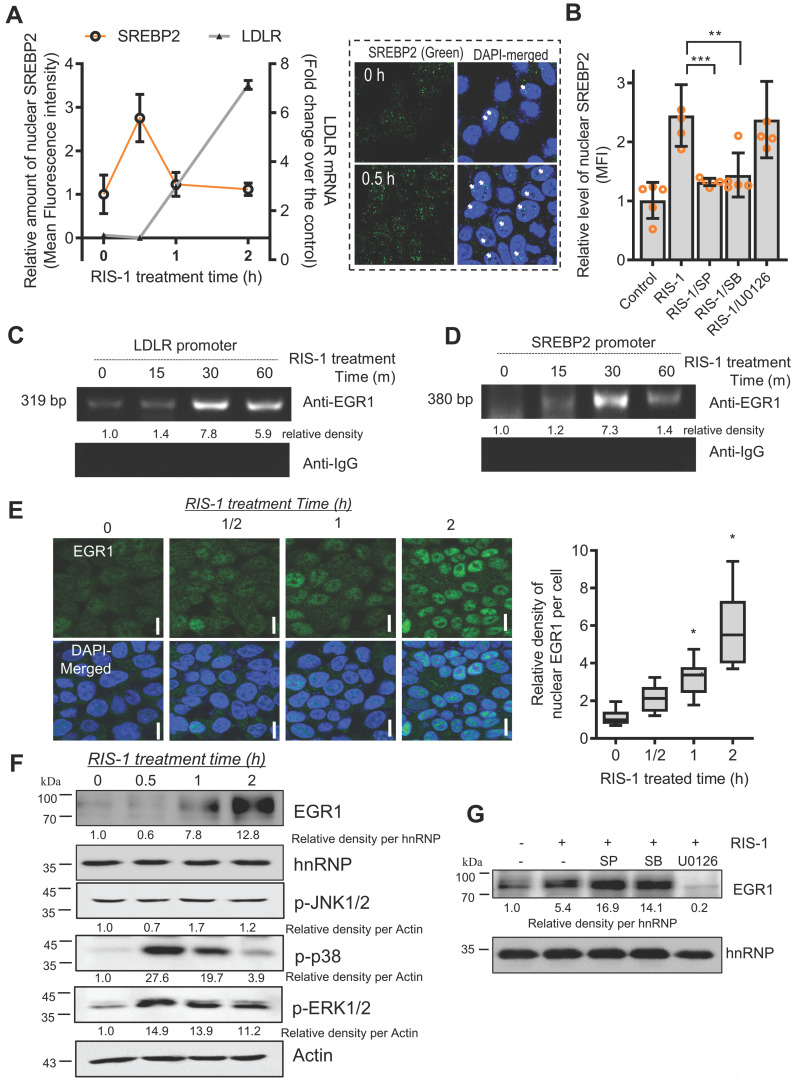
** LDLR expression in response to ribosomal inactivation is dependent on EGR1 and SREBP2.** (A) HCT-8 cells were treated with 1000 ng/mL RIS-1 for the indicated time. The graph shows the quantification of nuclear SREBP2 protein from the confocal microscopic analysis (boxed panels) and LDLR mRNA using reverse transcription-quantitative PCR (RT-qPCR). (B) HCT-8 cells were pretreated with the vehicle, 10 μM SP600125 (SP), 10 μM SB203580 (SB), or 2 μM U0126 for 2 h and then treated with vehicle or 1000 ng/mL RIS-1 for 30 min. The bar graph shows the quantification of nuclear SREBP2 protein using a confocal microscope (**p < 0.01 and ***p < 0.001). (C and D) For the ChIP assay, HCT-8 cells were treated with vehicle or 1000 ng/mL RIS-1 for the indicated time. (E) HCT-8 cells were treated with vehicle or 1000 ng/mL RIS-1 for the indicated time and examined under a confocal microscope. The microscopic analysis was performed at the original magnification of 1600×; scale bar(s), 20 μm. The right panel shows the relative quantitative values for nuclear EGR1 protein in cells. The relative ratio was obtained by measuring the nuclear density of EGR1 corresponding to the DAPI-stained area (the right graph, *p < 0.05). (F) HCT-8 cells were treated with vehicle or 1000 ng/mL RIS-1 for the indicated time. Nuclear fractions of cell lysates were subjected to western blot analysis. (G) HCT-8 cells pretreated with the vehicle, 10 μM SP600125 (SP), 10 μM SB203580 (SB), or 2 μM U0126 for 2 h were exposed to vehicle or 1000 ng/mL RIS-1 for 1 h. Nuclear fractions of cell lysates were subjected to western blot analysis. ChIP, chromatin immunoprecipitation; EGR1, early growth response 1; LDLR, low-density lipoprotein receptor; SREBP2, sterol regulatory element-binding protein 2.

To identify molecular mechanisms underlying LDLR-mediated intracellular cholesterol accumulation, we analyzed the role of transcription factors critical for LDLR expression in lipid accumulation in ribosome-inactivated intestinal epithelial cells. As shown in Figure 7A, the human LDLR promoter presented SRE-1 (+30 to +39) and sterol-independent regulatory element (SIRE; +75 to +94), which modulate LDLR transcription 36. SREBP2 preferentially activates gene transcription related to cholesterol synthesis and import, by specifically binding to SRE and plays a more dominant role* in vivo* than fatty acid synthesis-promoting SREBP1c 37. Moreover, given that the EGR1 protein is a putative transactivator of LDLR and SREBP2 promoters 36, 38, we postulated that EGR1 participates in LDLR-mediated regulation of both SRE-1 and SIRE (Figure 6A). To assess the involvement of SREBP2 and EGR1 in LDLR-associated cholesterol accumulation in response to ribosomal stress, we analyzed alterations in LDLR expression in EGR1- or SREBP2-deficient cells. Ribosomal stress-enhanced LDLR expression was significantly inhibited in EGR1- or SREBP2-deficient HCT-8 cells (Figure 6B), indicating the positive regulation of LDLR expression by transcription factors EGR1 and SREBP2. Mechanistically, we examined the transcriptional activation of the LDLR promoter-containing cis-elements for both transcription factors in cells with ribosomal inactivation. Ribosome inactivation enhanced the expression of the reporter gene linked to the LDLR promoter (+27 to +95), including both SRE and SIRE sites; however, a mutation in either SRE or SIRE (specifically, CRE) significantly reduced this expression (Figure 6C). In addition to the cis-elements, we assessed the effects of trans-regulatory elements on LDLR transcriptional activity. Genetic knockdown of EGR1 or SREBP2 using each shRNA remarkably downregulated the LDLR promoter activity in human intestinal cells (Figure 6D), supporting their involvement in LDLR transcription. Subsequently, ribosomal inactivation-induced cholesterol uptake was significantly diminished upon transfection of EGR1 and SREBP2 specific shRNA, as determined by Oil Red O staining (Figure 6E and 6F), suggesting that these transcription factors (SREBP2 and EGR1) are required for ribosomal stress-induced cholesterol regulation.

### EGR1 and SREBP2 bind to sterol-dependent and sterol-independent regulatory elements of the LDLR promoter in response to ribosomal stress

The SREBP2-associated LDLR promoter was assessed in ribosome-inactivated intestinal epithelial cells to examine transcriptional regulation of SRE-dependent LDLR expression. The microscopic analysis indicated that nuclear translocation of SREBP2 was transiently elevated by ribosomal dysfunction; subsequently, LDLR transcription was enhanced in ribosome-insulted intestinal epithelial cells (Figure 7A). Concerning the mechanism, given that MAP kinase-linked pathways extensively participate in ribosomal inactivation-linked cellular responses 28, we examined whether ribosomal stress-activated SREBP2 is dependent on the MAP kinase signaling pathway. Ribosomal stress-induced nuclear translocation of SREBP2 was significantly suppressed by inhibition of JNK1/2 or p38 MAP kinase, indicating that MAP kinase-linked signal transduction positively regulates SREBP2 activation (Figure 7B). Moreover, since both SREBP2 and LDLR promoters have cis-elements for EGR1 binding, we determined whether ribosomal stress can impact the association of EGR1 with SREBP2 and LDLR promoters via their consensus binding elements. As expected, ribosome inactivation enhanced the physical interaction of EGR1 with LDLR and SREBP2 promoters (Figure 7C and 7D, respectively). Next, we assessed the nuclear translocation of EGR1, given that EGR1 is also a crucial transcription factor for LDLR induction following ribosome inactivation. In addition to the chromatin immunoprecipitation assay (ChIP) assay results, EGR1 was found to be progressively and dose-dependently translocated into the nucleus, as demonstrated by the co-localization of EGR1 and DAPI on subcellular examination (Figure 7E). In addition, EGR1 expression was gradually increased in the nuclear fraction considering the time of ribosome inactivation (Figure 7F). As an upstream modulator of EGR1 expression, the mitogen-activated protein kinase (MAPK) signaling pathway was assessed in ribosome-insulted intestinal epithelial cells. Using specific pharmacological inhibitors of three key MAPK pathways, we demonstrated that ERK1/2 signaling is the main activator of ribosomal stress-induced EGR1 expression (Figure 7G). EGR1 accumulation in the nuclear compartment and its enhanced binding affinity to LDLR and SREBP-2, both responsible for lipid metabolism, appeared reminiscent of EGR1 involvement in cholesterol uptake triggered by ribosomal dysfunction. EGR1 knockdown attenuated the increase in fat droplet accumulation induced by ribosome inactivation (Figure 6E and 6F). In summary, the nuclear translocation and LDLR promoter binding of SREBP2 and EGR1 confirmed SRE- and SIRE-mediated LDLR transcription under ribosomal dysfunction.

### Post-transcriptional regulation of LDLR expression via human antigen R (HuR) protein

**Figure 8:**
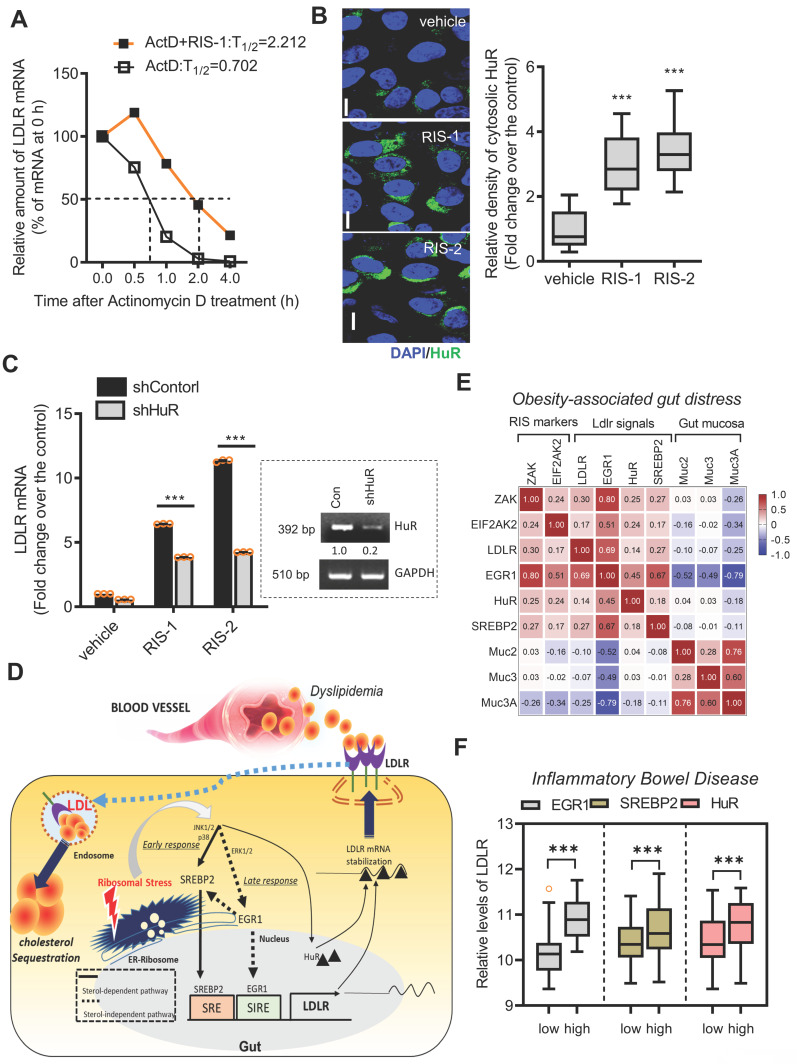
** Effects of ribosomal inactivation on LDLR mRNA stability in human intestinal cells.** (A) HCT-8 cells were treated with the vehicle or 1000 ng/mL RIS-1 for 2 h and then replaced with 5 μM actinomycin D for the indicated time to arrest cellular transcription. Expression levels of LDLR mRNA were measured using reverse transcription-quantitative PCR (RT-qPCR). The underlying boxed blots represent the mRNA measured by conventional RT-PCR. (B) HCT-8 cells were treated with vehicle, 1000 ng/mL RIS-1, or 2 μM RIS-2 for 2 h and examined using a confocal microscope. The microscopic analysis was performed at the original magnification of 1600×; scale bar(s), 20 μm. The right graph shows the relative quantitative values of cytosolic HuR in cells using ImageJ software (***p < 0.001). (C) HCT-8 cells transfected with the negative control vector or HuR shRNA expression vector (shHuR) were treated with the vehicle, 1000 ng/mL RIS-1, or 2 μM RIS-2 for 2 h. mRNA levels were measured using RT-qPCR. The boxed images show the suppression of HuR mRNA expression by shHuR (***p < 0.001). (D) A putative scheme for ribosomal inactivation-induced cholesterol uptake. (E) *Pearson*'s correlation analysis of relationships between transcription levels of critical components of ribosotoxic stress responses and LDLR in the small intestine of diet-induced obese mice (gse199776). Correlation matrix visualization was generated using the corrplot function of the R-package (R Foundation for Statistical Computing, Vienna, Austria. URL: https://www.R-project.org/). Correlations of transcriptional expression among genes were interpreted according to a general guideline for Pearson's coefficient value: r > 0.7, high (+); 0.5 < r < 0.7, moderate (+); 0.3 < r < 0.5, moderate (+) or low (+); 0.1 < r < 0.3, low (+); -0.1 < r < 0.1, negligible; -0.3 < r < -0.1, low (-). (F)* LDLR* expression was assessed in patients with IBD (gse75214; *Vemeire*'s, n = 194). Based on *EGR1, SREBP*, or *HuR* levels, we selected samples exhibiting the 50 highest and 50 lowest levels, which were further compared for *LDLR* levels. Results are shown as a box-and-whisker plot (Tukey), and asterisks (*) indicate significant differences from the low expression group (*p < 0.05, *** *p <* 0.001). EGR1, early growth response 1; HuR, human antigen R; IBD, inflammatory bowel disease; LDL, low-density lipoprotein; LDL, low-density lipoprotein receptor; SREBP, sterol regulatory element-binding protein.

In response to ribosomal stress, the maximal transcriptional activation of LDLR expression increased by less than 2-fold (Figure 6C-6D), whereas maximal levels of LDLR mRNA were enhanced by approximately 6-fold, indicating the presence of additional mechanisms underlying LDLR induction by ribosomal inactivation. Therefore, we assessed the post-transcriptional regulation of LDLR. Treatment with actinomycin D arrested cellular transcription, and the stability of the remaining LDLR mRNA was measured using quantitative PCR. Ribosomal stress enhanced the half-life of LDLR mRNA by more than 3-fold (Figure 8A). Mechanistically, the stability of LDLR mRNA is modulated via its 3' untranslated region (UTR), which contains four adenylate-uridylate (AU)-rich elements (AREs). Among ARE-binding proteins, the effect of HuR was examined on LDLR mRNA expression, given that ribosomal stress is known to promote the cytosolic translocation of HuR protein 39, 40. In human intestinal epithelial cells, ribosome inactivation significantly increased cytoplasmic HuR protein levels (Figure 8B). Moreover, when HuR expression was genetically downregulated using shRNA, LDLR mRNA levels were significantly reduced, indicating that HuR protein positively regulated LDLR expression (Figure 8C). Ribosomal insult promoted cytoplasmic translocation of HuR, which participated in LDLR mRNA stabilization.

Collectively, both transcriptional and post-transcriptional regulation contribute to LDLR induction via ribosome inactivation. SREBP2 and EGR1, pivotal transcription factors, were found to sequentially participate in LDLR induction, while EGR1 also positively regulated SREBP2 expression in ribosome-inactivated intestinal epithelial cells (Figure 8D). Moreover, stabilization of the LDLR transcript contributed to the upregulation of LDLR expression via the ARE-binding HuR protein. The mechanistic prediction of the ribosomal stress-induced event was verified in the metabolic disease model. The ribosomal stress-linked genes displayed positive correlations with the key signaling molecules (EGR1, SREBP2, and HuR) for LDLR induction despite negative correlations with gut mucin genes in the small intestine of diet-induced obese mice (Figure 8E). Consistent with the predictive results in the metabolic distress, patients with chronic colitis exhibiting high expression of pivotal signaling mediators such as EGR1, SREBP2, and HuR showed elevated levels of LDLR expression (Figure 8F), indicating a positive involvement of these signaling molecules in LDLR expression in IBD (Figure S7A-S7C).

## Discussion

Ribosomal stress has been predicted as an etiological factor in intestinal inflammatory diseases using diverse experimental models 23-25, 39, 41-43. Herein, we noted patients with ulcerative gut distress displayed enhanced levels of stress-responsive eIF2α kinases, the key signaling modules of the integrated stress responses. In particular, clinical transcriptome-based quantitation addressed the preferential elevation of EIF2AK2 rather than other eIF2α kinases. The EIF2AK2-associated gene cluster was markedly associated with the ribotoxic stress responses in IBD. Moreover, patients with ulcerative gut injuries tend to exhibit dyslipidemia, such as the dysregulation of circulating cholesterol. As a simulating model of metabolic dysfunction in patients with IBD, EIF2AK2-triggering chemical ribosomal stress was proven to facilitate enterohepatic sequestration of LDL cholesterol. Mechanistically, enterohepatic ribosomal stress led to tissue cholesterol uptake via modulation of EIF2AK2-induced LDLR. Ribosomes are reportedly a potential target for intervention against cholesterol-associated cardiovascular diseases 44-46. However, when ribosomal biogenesis was blocked, intracellular fat deposition was not detected (data not shown), suggesting the involvement of extraribosomal factors in ribosomal stress-induced fat accumulation. In the present study, ribosomal inactivation refers to the functional inhibition of mature ribosomal components. Considering the present results, ribosomal stress-associated ISR would be a promising signaling trigger leading to the extraribosomal signaling activation and subsequent regulation of genes involved in cholesterol uptake in response to ribosomal dysfunction.

LDLR transcription is largely controlled by a cholesterol-mediated negative feedback mechanism mediated via the interaction of SRE and SRE-binding proteins (SREBPs), whose nuclear translocation is controlled by intracellular cholesterol levels 47, 48. In addition to the intracellular cholesterol-dependent pathway, cumulative evidence from both *in vivo* and *in vitro* studies has identified SIRE, which mediates the stimulatory effects of cytokines, growth factors, hormones, and secondary messengers, such as cyclic AMP (cAMP), on LDLR transcription 49, 50. The SIRE motif (TGCTGTAAATGACGTGG) is located at +78 to +94, upstream of SRE-1 and the binding site of the transcription factor Sp1, and is composed of a C/EBP binding site (+78 to +86) and a cAMP-responsive element (CRE) site (+87 to +94) 51, 52. In particular, EGR1 is crucial for SIRE-mediated transcription of human LDLR via binding to CRE in the presence of cytokines, such as Oncostatin M 36. In the present ISR cell stress model, LDLR was associated with SREBP2/EGR1-activated transcriptional regulation, and to some extent, with post-transcriptional modulation. The stability of LDLR mRNA is modulated through the UTR, which contains four AREs 53, 54. Cytoplasmic ARE-binding proteins either destabilize bound transcripts or protect them against endonuclease cleavage. While heterogeneous nuclear ribonucleoprotein (HNRNP) D, HNRNPI, and KH-type splicing regulatory proteins act as decay-promoting factors that bind to ARE sequences in the 3'-UTR of LDLR, HuR can enhance the stability of LDLR mRNA 53, 54. As a well-established HuR trigger, ribosome inactivation promotes the cytosolic translocation of HuR protein, which can enhance the stability of ARE-containing transcripts of various early responsive genes, including cytokines and growth factors 39, 40. HuR protein shuttling across the nuclear membrane is regulated by different signaling molecules, including members of the MAPK family and protein kinase C (PKC) 39, 40. Ribosomal inactivation enhances the stability of pro-inflammatory cytokine mRNA via stress-activated signaling mediators such as p38 MAP kinase and PKC signaling pathways 55, 56. Accordingly, HuR translocation mediated by ribosomal insults could be associated with these signaling cascades, contributing to the stabilization of LDLR mRNA. In addition to the transcriptional regulation of LDLR, protein degradation of LDLR is an important determinant of maintaining the LDLR pool in the plasma membrane. Typically, circulating proprotein convertase subtilisin/kexin type 9 (PCSK9) forms an inhibitory binding complex with the catalytic domain of LDLR, which undergoes internalization into lysosomes for degradation 57. In particular, chemical-specific ribosomal stalling is known to inhibit the translation and secretion of PCSK9 as a key regulator of cholesterol receptors 44, 46, potently enhancing the surface bioavailability of LDLR protein owing to reduced PCSK9 expression. Moreover, PCSK9 mRNA expression was reduced by ribosomal stress in human gut cells despite elevations in LDLR levels (Figure S8). In the present study, we focused on early stress-responsive gene regulation via transcriptional and post-transcriptional regulation. In addition to the early stress-responsive gene regulation, chronic processes such as epigenetic regulation may contribute to lipid metabolism-linked gene expression. In particular, DNA demethylation using 5-Azacytidine (5-AZA, a DNA methyltransferase inhibitor, Figure S8A-S8B), histone demethylation using GSK126 (a selective inhibitor of EZH2 methyltransferase, Figure S8C-S8D), or histone acetylation using trichostatin A (TSA, a histone deacetylase inhibitor, Figure S8E-S8F) partly counteracted PCSK9 reduction and LDLR induction in response to ribosomal stressors. It is thus warranted to assess chronic actions of ribosomal stress in epigenetic reprogramming in the next studies based on the present evidence for prolonged stress-responsive molecular events.

In the present study, fat bodies were attributed to the increased uptake of extracellular lipids via LDLR; however, we cannot rule out the possibility that fat droplets may be due to increased triglyceride (TG) accumulation, which can be caused by a defect in any of the steps from synthesis to release of TG conjugated with apoproteins. Considering cytopathological events, malnutrition and tissue damage induced by toxic chemicals are known to decrease apoprotein synthesis, resulting in fat accumulation in the liver 58, 59. Clinically, cholesterol accumulation plays an important role in the progression of nonalcoholic fatty liver diseases, such as nonalcoholic steatohepatitis (NASH) 60. Although lipid accumulation and subsequent tissue injuries by ribosomal stress have been reported in fish and avian models 61-63, we did not observe the previously reported intracellular fat accumulation-linked cytotoxic patterns in the present stress model. Instead, ribosomal stress-induced LDLR expression may be associated with other chronic actions such as tumorigenesis since lipid accumulation and expression of LDLR are elevated in intestinal polyps of patients with cancer or animal models of cancer, compared with those of normal colonic mucosa 64-66. Mechanistically, it has been speculated that LDLR-linked signaling pathways, including phosphoinositide 3 kinase-mediated signals or elevated arachidonic metabolism from the pool of fatty acids, could play a pivotal role in the growth of human colon cancer cells and subsequent polyp formation 64-66. LDLR may be overexpressed in areas where lipid droplets can be observed; in particular, the expression levels of LDLR mRNA in the intestinal polyps of Min mice were shown to be higher than those in the non-tumorous parts 66. Altered arachidonic metabolism can be associated with the notable expression of cyclooxygenase-2, mainly distributed in stromal cells and expressed in epithelial cells 66. Ribosome inactivation can directly induce cyclooxygenase-2 via MAPKs and trigger pro-oncogenic arachidonic metabolites 28. Therefore, LDLR-mediated lipid uptake and arachidonic acid metabolism may participate in malignant processes in ribosome-insulted transformed epithelia. Although enterohepatic LDLR expression is valuable for lowering blood cholesterol levels, prolonged activation of LDLR-linked signaling must be carefully addressed in chronic stress-insulted gastrointestinal malignancies.

In conclusion, patients with inflammatory intestinal distress displayed enhanced levels of ribosomal stress-responsive eIF2α kinase, which was notably associated with lipid metabolic process genes. Eukaryotic ribosomes were assessed as a novel stress-responsive module for metabolic homeostasis. Although ribosomal stress caused extensive alterations in cellular lipid metabolic gene profiles, cholesterol import-associated pathway genes such as the low-density lipoprotein receptor (*LDLR*) were remarkably elevated, which contributed to enterohepatic fat sequestration and lowering circulatory LDL cholesterol. In addition to the chemical-based experimental model, the ribotoxic stress response was associated with expressions of genes for LDLR and LDLR-enhancing signaling modules in the gut of subjects with human inflammatory bowel diseases (IBD) or diet-induced obesity. The simulated ribosome-linked assessment of cellular reprogramming provides predictive insights into stress-responsive regulations of gut lipid homeostasis during human chronic distress.

## Materials and Methods

### Cell culture and chemicals

Human epithelial cell lines including intestinal HCT-8 and hepatic HepG2 were supplied by the American Type Culture Collection (ATCC; Rockville, MD, USA). Cells were maintained in RPMI 1640 medium, supplemented with 10% (v/v) heat-inactivated fetal bovine serum (FBS), 50 U/mL penicillin, and 50 mg/mL streptomycin, all of which were purchased from Welgene (Daegu, Korea)], in a 5% CO_2_ humidified incubator at 37 °C. Deoxynivalenol (RIS-1; purity, 97.6 ± 2.4%) was isolated from *Fusarium graminearum*, and anisomycin (RIS-2; purity, 98%) was isolated from *Streptomyces griseolus* (Sigma-Aldrich, St. Louis, MO, USA). MAPK inhibitors, including SP600125 (SP, a JNK1/2 inhibitor), SB203580 (SB, a p38 inhibitor), U0126 (a MEK1/2 inhibitor), 5-Azacytidine (5Aza, a DNA methyltransferase inhibitor), and GSK126 (an EZH2 methyltransferase inhibitor) were purchased from Sigma-Aldrich. Trichostatin A was purchased from Wako Pure Chemical Industries (Osaka, Japan).

### Mouse experiments

Three- to ten-week-old male C57BL/6J mice were purchased from Samtako Bio Korea (Osan, Korea), and male C57BL/6J, LDLR-KO mice (B6.129S7-Ldlr^tm1Her^/J) were kindly provided by Dr. Goo Taeg Oh (Ewha Woman's University, Seoul, Korea). To induce ribosome-inactivating stress in the gastrointestinal tract, six- or ten-week-old mice were exposed to RIS-1 or RIS-2 at 25 mg/kg body weight, based on the previous report 23-25. To establish the high-fat diet (HFD) model, three-week-old male C57BL6J mice were fed an AIN-76A-based cereal feed (#100000, Dyets Inc., Bethlehem, PA, USA) or 60 kcal% saturated fat (lard) diet (D12492, Research Diets Inc., New Brunswick, NN, USA) for 12 weeks.

### Ethical approval

All animal experiments and care were approved by the Institutional Animal Care and Use Committee of Pusan National University (PNU-2010-0291), and the methods were performed under the Declaration of Helsinki and the Guide for the Care and Use of Laboratory Animals as adopted and promulgated by the United States National Institutes of Health.

### Histological analysis

H&E staining was performed using standard procedures. Briefly, small intestine paraffin blocks were sectioned at 5 mm, and then dehydration, sections were stained with hematoxylin and eosin (H&E) at room temperature according to the previously reported methods (hematoxylin staining for 5 min and eosin staining for 2 min). Scoring of small intestinal injury was based on the following four parameters. Inflammatory cell infiltration was scored as follows: 0, rare inflammatory cells in the lamina propria; 1, increased numbers of granulocytes in the lamina propria; 2, confluence of inflammatory cells extending into the submucosa; and 3, transmural extension of the inflammatory infiltrate. Crypt damage was scored as follows: 0, intact crypts; 1, loss of the basal half; 2, entire crypt loss; and 3, confluent erosion. Ulceration was scored as follows: 0, absence of ulcer; 1, one or two foci of ulcerations; 2, three or four foci of ulcerations; and 3, confluent or extensive ulceration.

### Mouse intestinal organoid culture

Small intestinal crypts were isolated from 12-20-week-old mice. Then, cut tissue pieces were incubated in cell dissociation reagent (STEMCELL Technologies, Vancouver, BC, Canada) for 15 min, and isolated crypts, in 50 μl Matrigel, were seeded in each well of a 24-well plate of the following culture medium mixture. The L-WRN cell line (ATCC)-conditioned medium was mixed with an equal volume of basal crypt media (advanced DMEM/F12 (ADF), supplemented with Glutamax (2 mM, Gibco, Thermo Fisher Scientific, Inc., Waltham, MA, USA), penicillin (100 U/mL), streptomycin (0.1 mg/mL), N2 supplement (1X, Gibco), B27 supplement (1X, Gibco), human epidermal growth factor (EGF) (50 ng/mL; Peprotech, Rocky Hill, NJ, USA), and NAC (1 mM; Sigma-Aldrich). After ten days, differentiated small intestine organoids were exposed to RIS.

### Oil Red O staining

In brief, cells were washed in phosphate-buffered saline (PBS) and incubated in 10% paraformaldehyde for 30 min at room temperature. After washing three times in PBS, cells were incubated in 60% isopropanol for 5 min at room temperature, followed by three washes using PBS and incubation in Oil Red O solution for 10 min at room temperature. Finally, the cells were washed thrice with deionized water. Images were captured using a microscope. For tissue staining, tissue samples were fixed in ice-cold 10% formalin for 10 min at room temperature. After washing tissue thrice in distilled water, samples were incubated in absolute propylene glycol for 5 min at room temperature and treated with Oil Red O solution for 1 h (small intestine) or 10 min (liver) at 60 °C. Tissue slices were further incubated in an 85% propylene glycol solution for 3 min at room temperature, followed by three washes in distilled water. Finally, samples were counterstained with hematoxylin for 10 min. Mouse small intestine organoid samples were washed with media, followed by the addition of Corning® Cell Recovery Solution for 1 h at 4 °C. After fixing in 4% paraformaldehyde in PBS for 1 h on ice, tissues were stained with Oil Red O solution (0.18% Oil Red O in 60% isopropanol) for 20 min at room temperature. Images were captured using a microscope (ECLIPSE Ts2R-FL, Nikon, Tokyo, Japan).

### Filipin staining

Cells and tissues were stained to detect free cholesterol using commercially available Filipin III (Sigma-Aldrich), according to the manufacturer's instructions. Slides were fixed with 4% paraformaldehyde for 30 min, washed with PBS, and incubated with freshly prepared filipin solution (100 μg/mL) for 1 h in the dark. The slides were then washed with PBS, and a drop of glycerol in PBS (glycerol: PBS = 1:9) was added. The samples were visualized using an ECLIPSE Ts2R-FL inverted microscope (Nikon, Tokyo, Japan).

### Cell-based LDL uptake assay

Cells were assessed for LDL uptake using a commercially available LDL uptake cell-based assay kit (Cayman Chemical Company, Ann Arbor, MI, USA), according to the manufacturer's instructions. Briefly, HCT-8 cells were seeded in 96-well plates at a density of 3 × 10^4^ cells per well. After 48 h, cells were treated with 1000 ng/mL RIS or 2 μM RIS-2 for 24 h. At the end of the treatment period, cells were incubated with LDL conjugated to DyLight™ 550 for 4 h and visualized using an AXIO Imager M2 fluorescence microscope (Carl Zeiss, Jena, Germany) at excitation and emission wavelengths of 540 and 570 m, respectively. Following measurement of LDL uptake, cellular LDLR was detected using a rabbit anti-LDL receptor antibody.

### Cellular lipid analysis

For intracellular cholesterol and TG analyses, samples were prepared using the Cholesterol/Cholesteryl Ester Quantification kit (Biovision, San Diego, CA, USA), according to the manufacturer's instructions. Total cholesterol was measured using a Multilabel plate reader (VICTOR3, Perkin Elmer, Waltham, MA, USA) at excitation and emission wavelengths of 535 and 587 nm, respectively. Protein lysates were quantified using a BCA kit (Pierce, Rockford, IL, USA). For the cellular TG assay, the cells were lysed in 200 μl of 1% Triton X-100 by sonication for 30 s and then centrifuged at 10,000×*g* for 20 min at 4 °C. The supernatants were assayed for total TG using a commercially available TG-S reaction kit (Asan Pharm. Co., Seoul, Korea) according to the manufacturer's instructions. Measurements were performed using a microplate reader (VersaMax, Molecular Devices, Sunnydale, CA, USA) at 550 nm. Protein lysates were quantified using a BCA protein assay kit (Pierce). To measure cholesterol and TG levels, blood samples were mixed with 5 mM EDTA (pH 8.0) and centrifuged at 1,000×*g* for 15 min at 4 °C. The supernatant serum samples were transferred to new tubes and centrifuged at 10,000×*g* for 10 min at 4 °C. Plasma samples were stored at -80 °C until use. The amounts of cholesterol and triglycerides (TG) were measured using FUJI DRI-CHEM slides (FUJIFILM, Tokyo, Japan) with FUJI DRI-CHEM 3500I (FUJIFILM). The cellular lipidomic profile was assessed using mass spectrometry-based lipid analysis performed by Lipotype GmbH (Dresden, Germany) as previously described 67. Lipids were extracted using chloroform and methanol, and extracted samples were spiked with lipid class-specific internal standards before extraction. After drying and resuspending the mass spectrometry acquisition mixture, lipid extracts were subjected to mass spectrometric analysis. The samples were analyzed using a Lipotype shotgun lipidomics platform. The identified lipid molecules were quantified by normalization to a lipid class-specific internal standard. The amounts of individual lipid molecules (species or subspecies) of a given lipid class were summed to yield the total amount of the lipid class. The following is a list of lipid abbreviations: cholesterol esters (CE), diacylglycerol (DAG), phosphatidylcholine (PA), phosphatidylcholine (PC), phosphatidylethanolamine (-ether, PE), phosphatidylglycerol (PG), phosphatidylinositol (PI), phosphatidylserine (PS), sphingomyelin (SM), and triacylglycerol (TAG).

### Immunohistochemistry

Mouse small intestinal tissues were dehydrated, embedded in paraffin, and cut into 5-μm thick sections for immunohistochemical analysis. The sections were examined at various magnifications using an AXIO Imager M2 (Carl Zeiss AG, Oberkochen, Germany). Images of normal tissue and lesions were captured and the target protein signaling was measured by computer-assisted analysis using Histo-quest software 4.0 (TissueGnostics GmbH, Vienna, Austria).

### Confocal microscopy

Cells were assayed by confocal microscopy, as previously described 39. Briefly, cells were incubated with rabbit polyclonal anti-human EGR1 antibody (1:500), rabbit polyclonal anti-human SREBP2 (1:200), mouse monoclonal anti-human HuR antibody (1:200; Santa Cruz Biotechnology), and rabbit polyclonal anti-human LDLR antibody (1:200; Cayman Chemical, Ann Arbor, Michigan, USA) in buffer (3% bovine serum albumin in PBS) for 2 h at room temperature. The cells were then washed in PBS, incubated with FITC-conjugated goat anti-rabbit IgG (H+L; Invitrogen) for 2 h at room temperature, washed in PBS, and stained with 100 ng/mL DAPI (absorbance at 405 nm) in PBS for 10 min.

For organoid immunofluorescence staining, organoids were disaggregated by one-hour incubation in the cell recovery solution (Corning, NY, USA) at 4 °C and then fixed with PBS containing 4% paraformaldehyde overnight at 4 °C. After washing with PBST, the intestine organoids were incubated with PBS containing 1 % Triton X-100 for 30 m at 25 °C, followed by treatment with the primary antibody against LDLR (1:200, ABclonal, Woburn, MA, USA) overnight at 4 °C and another two-hour incubation with the secondary antibody (fluorescein isothiocyanate (FITC)-conjugated goat anti-rabbit IgG (H+L; Invitrogen)) at 25 °C. The intestinal organoids were counterstained with a DAPI mounting solution (IHC-Tek^TM^ DAPI solution, IHC WORLD, Woodstock, MD, USA) and images were acquired using an Andor's BC43 benchtop confocal microscope (Oxford Instruments, Belfast, UK).

### Electron microscopy

For TEM, cells were collected 24 h after RIS-1 or RIS-2 treatment and fixed with 2% glutaraldehyde (in 0.1M sodium cacodylate buffer, pH 7.2) for 2 h at 4 °C. The cells were washed three times with cacodylate buffer and gently scraped off the dishes. The pelleted cells were embedded in 2% agarose prepared in water. The agarose block was post-fixed for 20 min with 1% osmium tetraoxide in 0.1 M phosphate buffer (pH 7.4) and washed three times with cacodylate buffer. The agarose block was then stained with 0.5% uranyl acetate (in 50% MeOH), washed with cacodylate buffer, and dehydrated using graded alcohol concentrations (30%, 50%, 70%, 95%, and 100%) and propylene oxide. Sections were obtained using an ultramicrotome (Leica Microsystems, Wetzlar, Germany), mounted on Formvar-coated grids, and stained with uranyl acetate. Images were captured with a Hitachi H7600 transmission electron microscope (Hitachi, Tokyo, Japan).

### Construction of plasmids and transfection

Cytomegalovirus (CMV)-driven shRNA was constructed by inserting the shRNA template into the pSilencer 4.1-CMV neo vector (Ambion, Austin, TX, USA). The negative control vector and shRNA of SREBP2, EGR1, HuR, and LDLR were denoted Con, shSREBP2, shEGR1, shHuR, and shLDLR, respectively. The negative control siRNA template sequence lacks significant homology to the mouse, human, and rat genome databases, and pSilencer 4.1-CMV neo containing the negative control siRNA template was provided by the Ambion Company. SREBP2, EGR1, HuR, and LDLR shRNA targeted the sequence 5'-GGC TTT GAA GAC GAA GCT A-3', 5'-GTT ACT ACC TCT TAT CCA T-3', 5'-GTG CAA AGG GTT TGG CTT T-3', and 5'-GGA CAG ATA TCA TCA ACG A-3', respectively. The human LDLR promoter (+94 to +30)-containing plasmids (pLDLR234-WT, pLDLR234-SRE-mutant, and pLDLR 234-CRE-mutant) were kindly provided by Dr. Jingwen Liu (Department of Veterans Affairs, Palo Alto Health Care System, Livermore, CA, USA). For transient expression of shRNA, cells were transfected using jetPRIME transfection reagent (Polyplus-transfection, New York, NY, USA) according to the manufacturer's instructions. For transfection of the luciferase reporter gene, a mixture of 2 μg firefly luciferase reporter and 0.2 μg *Renilla* luciferase, pRL-null vector (Promega, Madison, WI, USA) per 2 μl of OmicsFect^TM^ reagent (Omics Biotechnology, Taiwan) was applied to each well of a 6-well culture plate. To perform the luciferase assay, 12 h after transfection, cells were exposed to chemicals for an additional 24 h, lysed, and assayed using the Dual-Luciferase Reporter Assay System (Promega).

### Quantitative PCR

RNA was extracted using RiboEX (GeneAll Biotechnology, Seoul, Korea) according to the manufacturer's instructions. RNA (100 ng) from each sample was transcribed into cDNA using Prime Moloney murine leukemia virus reverse transcriptase (Genetbio, Nonsan, Korea). Amplification was performed using n-Taq DNA polymerase (Enzynomics, Seoul, Korea) on a MyCycler thermal cycler (Bio-Rad, Hercules, CA, USA). PCR was performed using the forward- and reverse-complement PCR primers (Table 1) under the following parameters: denaturation at 95 °C for 2 min, different cycles of denaturation at 95 °C for 30 s, annealing at 58 °C for 30 s, and elongation at 72°C for 30 s. An aliquot of each PCR product was subjected to 1% (w/v) agarose gel electrophoresis and visualized using ethidium bromide staining. For real-time PCR, FAM was used as the fluorescent reporter dye and was conjugated to the 5′ ends of probes to detect amplified cDNA in an iCycler thermal cycler (Bio-Rad, Hercules, CA, USA) using the following parameters: denaturation at 94 °C for 2 min and 40 cycles of denaturation at 98 °C for 10 s, annealing at 59 °C for 30 s, and elongation at 72 °C for 45 s. Each sample was tested in triplicate. The relative gene expression was quantified using the comparative threshold cycle (CT) method. The CT value was defined as the point at which a statistically significant increase in fluorescence occurred. The number of PCR cycles (CT) required for the FAM intensity to exceed a threshold just above the background was calculated for test and reference reactions. GAPDH was used as the endogenous control in all experiments.

### Western blot analysis

Protein expression levels were determined using western blot analysis. In brief, 50 µg of protein was separated by mini gel electrophoresis (Bio-Rad) and transferred onto a PVDF membrane (Pall Corporation, New York, NY, USA). The membranes were then incubated with the following antibodies: rabbit polyclonal anti-human actin antibody (1:1000), rabbit polyclonal anti-human SREPB2 antibody (1:1000), rabbit polyclonal anti-human EGR1 antibody (1:1000), mouse monoclonal anti-human HuR antibody (1:2000), mouse monoclonal anti-human hnRNP antibody (1:2000) (all from Santa Cruz Biotechnology, Santa Cruz, CA, USA), and rabbit polyclonal anti-human LDLR antibody (1:1000) (Cayman Chemical, Ann Arbor, Michigan, USA) for 2 h at room temperature.

### Chromatin immunoprecipitation (ChIP)

ChIP analysis was performed as previously described 24. The 5′ forward and 3′ reverse-complement PCR primers for the amplification of each gene were as follows: human LDLR promoter (5'-TTC AGA AGA TGC GTT TCC AA-3' and 5'-TCA CGA CCT GCT GTG TCC TA-3') and human SREBP2 promoter (5'-CAA ATC TGA GCT GCT GAT CG-3' and 5'-GTG AGG GTC TCC ATG GTC TC-3').

### Luciferase assay

Briefly, cells were washed with cold PBS, lysed with passive lysis buffer (Promega), and centrifuged at 12,000*×g* for 10 min. The supernatant was collected and stored at -80 °C until the assessment of luciferase activity. Luciferase activity was measured using a model TD-20/20 dual-mode luminometer (Turner Designs, Sunnyvale, CA). Firefly luciferase activity was normalized against *Renilla* luciferase activity by dividing firefly luciferase activity by *Renilla* luciferase activity.

### Analysis of intestinal mRNA of patients with IBD

Human colonic tissue datasets were obtained from gene expression arrays of patients with IBD (gse117993 [Denson's, n = 190], gse10616 [G, Sleiman's, n = 58], and gse75214 [Vemeire's, n = 194]). For the clinical dataset, the three major clinical subsets of IBD included only cCD, iCD, and UC. These experiments tested differential colonic gene expression in these three types of IBD relative to healthy control samples

### Single-cell RNA-sequencing (scRNA-Seq) analysis of intestinal transcriptome

All data analyses of single cell-based sequencing studies were performed with version R v4.1.3. The scRNA-Seq analysis was performed using the Seurat R package v4.1.0, with the batch effect of the data eliminated. The UMAP algorithm was adopted for nonlinear dimension reduction of the scRNA-Seq data. Marker genes of each cell cluster were found with the FindAllMarkers function. Cell clusters were annotated using the SCINA R package v1.2.0 (https://github.com/jcao89757/SCINA).

### Statistical analysis

Statistical analyses were performed using GraphPad Prism v. 5.01 (La Jolla, CA, USA). For comparative analysis of two groups of data, the Student's *t*-test was performed. For comparative analysis of multiple groups, data were subjected to analysis of variance (ANOVA) with the *Newman-Keuls* method as a *post hoc* ANOVA assessment. For two gene correlation coefficient (R) determination, *Pearson*'s correlation analysis was performed.

## Supplementary Material

Supplementary figures.

## Figures and Tables

**Table 1:** The forward- and reverse-complement PCR primers for amplification of each gene

Gene	Forwad primer sequence	Reverse primer sequence
h-GAPDH	5′- TCA ACG GAT TTG GTC GTA TT -3′	5′-CTG TGG TCA TGA GTC CTT CC-3′
h-LDLR	5′-CAG GAG ACA TCC ACC GTC AG-3′	5′-TAG CTG TAG CCG TCC TGG TT-3′
h-EGR1	5′-CAG TGG CCT AGT GAG CAT GA-3′	5′-CCG CAA GTG GAT CTT GGT AT-3′
h-SREBP2	5′-AAG TCT GGC GTT CTG AGG AA-3′	5′-CAC AAA GAC GCT CAG GAC AA-3′
h-ApoA1	5′- ATG AAA GCT GCG CTG CTG AC-3′	5′- ACC TCC TCC AGA TCC TTG CT-3′
h-ApoB	5′- GCC ATT GCG ACG AAG AAA ATA -3′	5′-TGA CTG TGG TTG ATT GCA GCT T-3′
h-ApoC2	5′-AAG ATG AGA TGC CTA GCC CG-3′	5′-ACT CCT CTC CCT TCA GCA CAG-3′
h-ApoC3	5′-CCC GGG TAC TCC TTG TTG TT-3′	5′-GGA ACT GAA GCC ATC GGT CA-3′
h-ApoE	5′-CAC TGG GTC GCT TTT GGG AT-3′	5′-GCA CAC GTC CTC CAT GTC C-3′
h-Fasn	5′- AGG CCT CAT AGA CCT GCT GA-3′	5′- TTG GCA AAC ACA CCC TCC TT-3′
h-Acaca	5′- CAT CTC CCT TGG CCC AAC C -3′	5′-GCTGGA GAA GCC ACA GTG AA-3′
h-PPARγ	5′-TTC AGA AAT GCC TTG CAG TG-3′	5′-CAC CTC TTT GCT CTG CTC CT-3′
h-PPARα	5′-AAG AGT AGC TTG GAG CTC GG-3′	5′-TGA AAG CGT GTC CGT GAT GA-3′
h-Acat1	5′-GGA GGC TGG TGC AGG AAA TA-3′	5′-CCC AAT ACT GCC TGC CTT GT-3′
h- cox1	5′- AAC TCA CCT TCG AGG CTT GG-3′	5′- TCC TGG CAA AGG CTT ATG GG-3′
h-Cpt1a	5′- ATG TAC GCC AAG ATC GAC CC-3′	5′-AGG CCT CAC CGA CTG TAG AT-3′
h-FASN	5′-ATC ATT GGG CAC TCC TTG GG-3′	5′-GGG GGC AAT TCC TTC CAT GA-3′
h-Abca1	5′-CTG CTA AGG AGG GAG CCT TT-3′	5′-AAA AGG GCC ACA AAC TGT TG-3′
h-NPC1L1	5′-GTT GCA ATG AGT CCC AAG GT-3′	5′-ACC GGG ATG ACA GAT AGC AC-3′
m-SREBP1	5′-CAG TGG CCT AGT GAG CAT GA-3′	5′-CCG CAA GTG GAT CTT GGT AT-3′
m-ACACA	5′-GGA GCG GGA GGA GTT CCT AA-3′	5′-CTA TCA CAG AGC GGA CGC CA-3′
m-PPARα	5′-TCT GGG CAA GAG AAT CCA CG-3′	5′-CAA GGA GGA CAG CAT CGT GA-3′
m-ACAT1	5′-CGC TCT CAG CTC TTA CAC CA-3′	5′-TCT ACG GCA GCA TCA GCA AA-3′
m-ACOX1	5′-ACT ACC TGG ACA GCC AAT GC-3′	5′-TCT ACC AAT CTG GCT GCA CG-3′
