# Supplementary material for: Gut ribotoxic stress responses facilitate dyslipidemia via metabolic reprogramming: an environmental health prediction
Source: Theranostics. 2024 Jan 20;14(3):1289–311. doi: 10.7150/thno.88586 (PMC10845207; doi:10.7150/thno.88586)
Supplement: Supplementary file 1 — Supplementary figures. [file thnov14p1289s1.pdf]

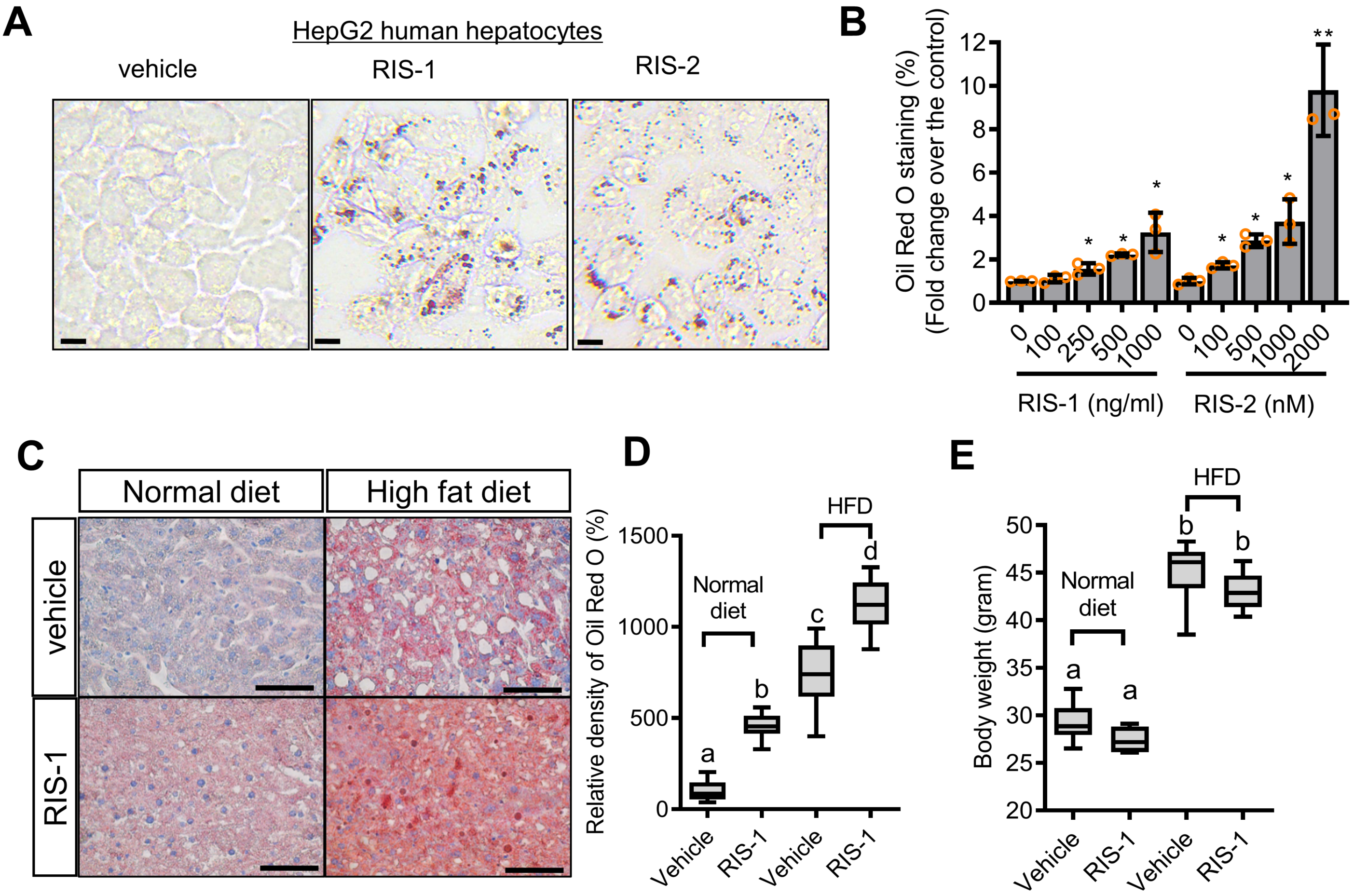

Figure S1. Ribosomal stress-associated fat deposition in the liver. (A-B) HepG2 cells were treated with the vehicle, 1000 ng/mL RIS-1, or 2 μM RIS-2 for 24 h. Intracellular lipid droplets were stained with Oil Red O and quantified using the ImageJ software. The graph indicates dose responses of the lipid droplet levels, and the asterisks represent a significant difference from each vehicle control (B, *$p < 0.05$ and **$p < 0.01$). The microscopic analysis was performed at the original magnification of 200×; scale bar(s), 50 μm. (C-E) Three-week-old C57 BL6J mice were fed normal chow or 60% high-fat diet (HFD) from weaning for 12 weeks. Mice were then treated with the vehicle or 25 mg/kg RIS-1 (25 mg/kg) for 24 h via oral gavage. Representative Oil Red O staining of the liver at the original magnification of 400×; scale bar(s), 50 μm (C). Relative quantitative values of Oil Red O-positive lipid droplets were quantified using ImageJ software (D). Body weight were also measured (E). Results are shown as box-and-whisker plots (Tukey), and different letters over each box represent significant differences between groups ($p < 0.05$ using one-way ANOVA with the Newman–Keuls post hoc test).

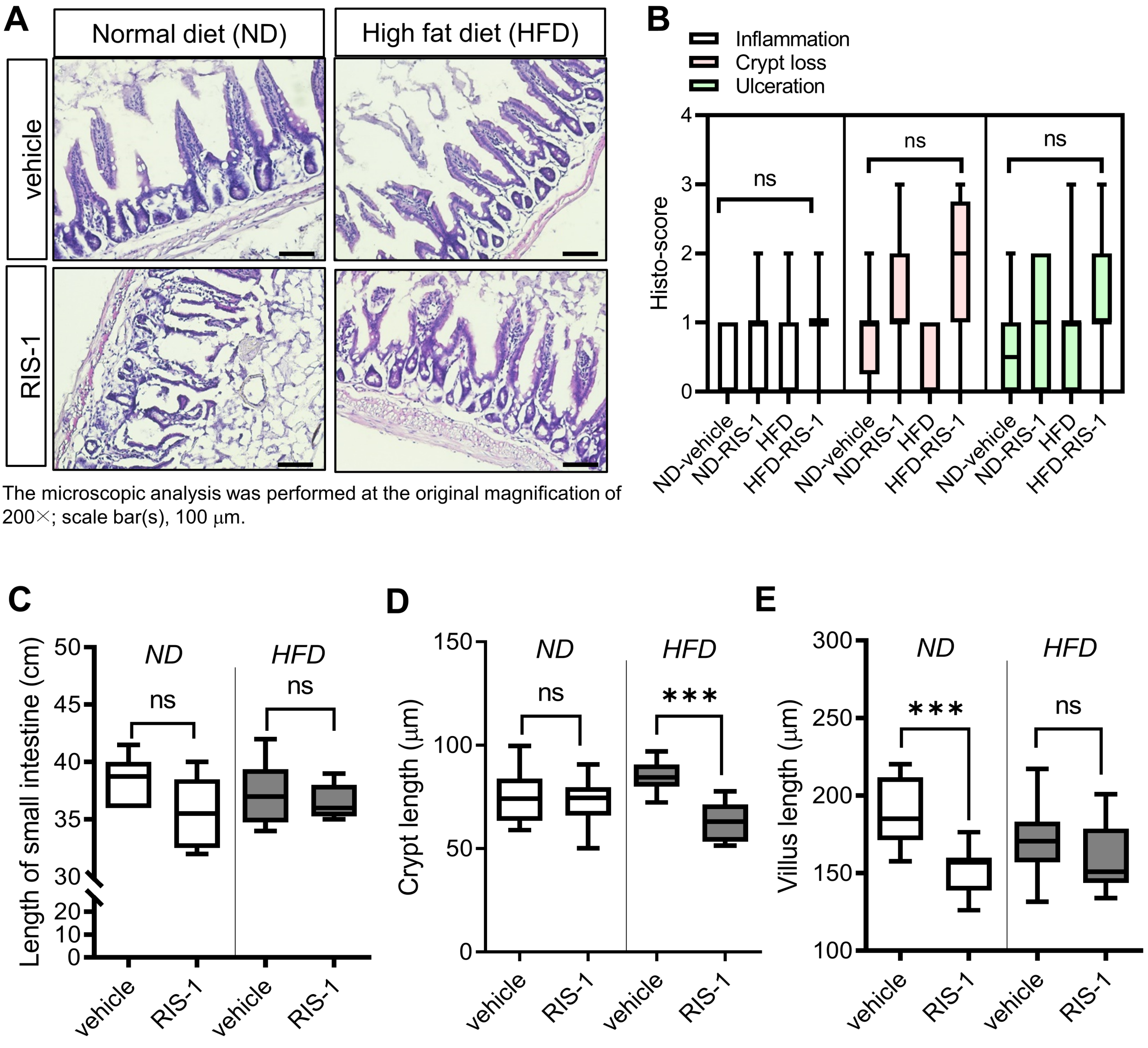

Figure S2. Ribosomal stress-associated cholesterol deposition in the gut and liver. Three-week-old C57 BL6J mice were fed normal chow or 60% high-fat diet (HFD) from weaning for 12 weeks. Mice were then treated with the vehicle or 25 mg/kg RIS-1 (25 mg/kg) for 24 h via oral gavage. Representative H&E staining of the small intestine (A, original magnification of 200×; scale bar (s), 100 μm) and histopathological scores (B). (C) The length of the small intestine was measured at the end of the experiment. (D-E) The length of the crypt (D) or villus (E). Results are shown as box-and-whisker plots (Tukey), and the asterisks represent a significant difference between groups. "*"*p*<0.05, "**"*p*<0.01, "***"*p*<0.001.

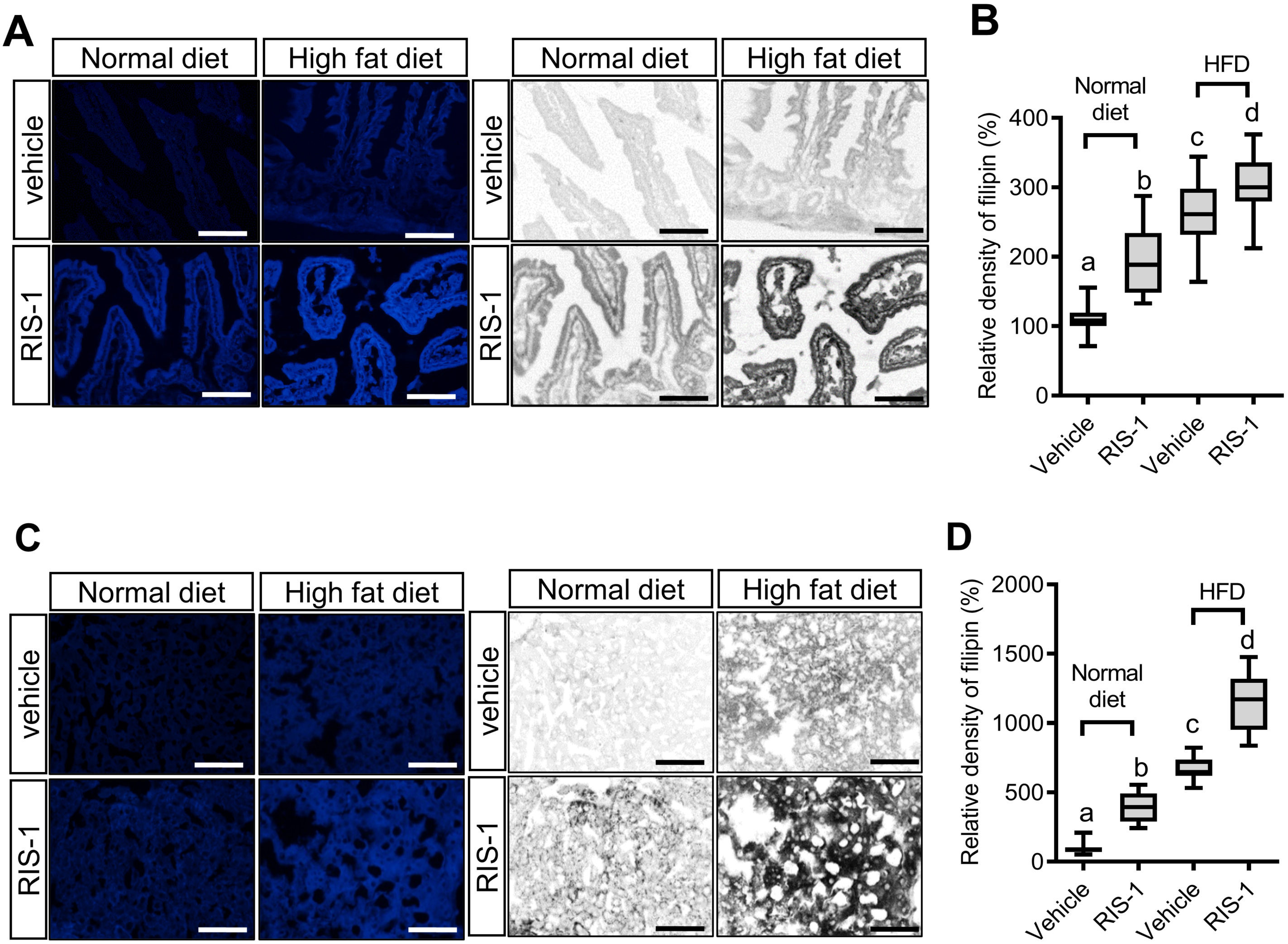

Figure S3. Ribosomal stress-associated cholesterol deposition in the gut and liver. Three-week-old C57 BL6J mice were fed normal chow or 60% high-fat diet (HFD) from weaning for 12 weeks. Mice were then treated with the vehicle or 25 mg/kg RIS-1 (25 mg/kg) for 24 h via oral gavage. Relative levels of fluorescence in the gut (A) and liver (C) were quantified using ImageJ software (B and D, respectively). Results are shown as box-and-whisker plots (Tukey), and different letters over each box represent significant differences between groups ($p < 0.05$ using one-way ANOVA with the Newman–Keuls post hoc test).

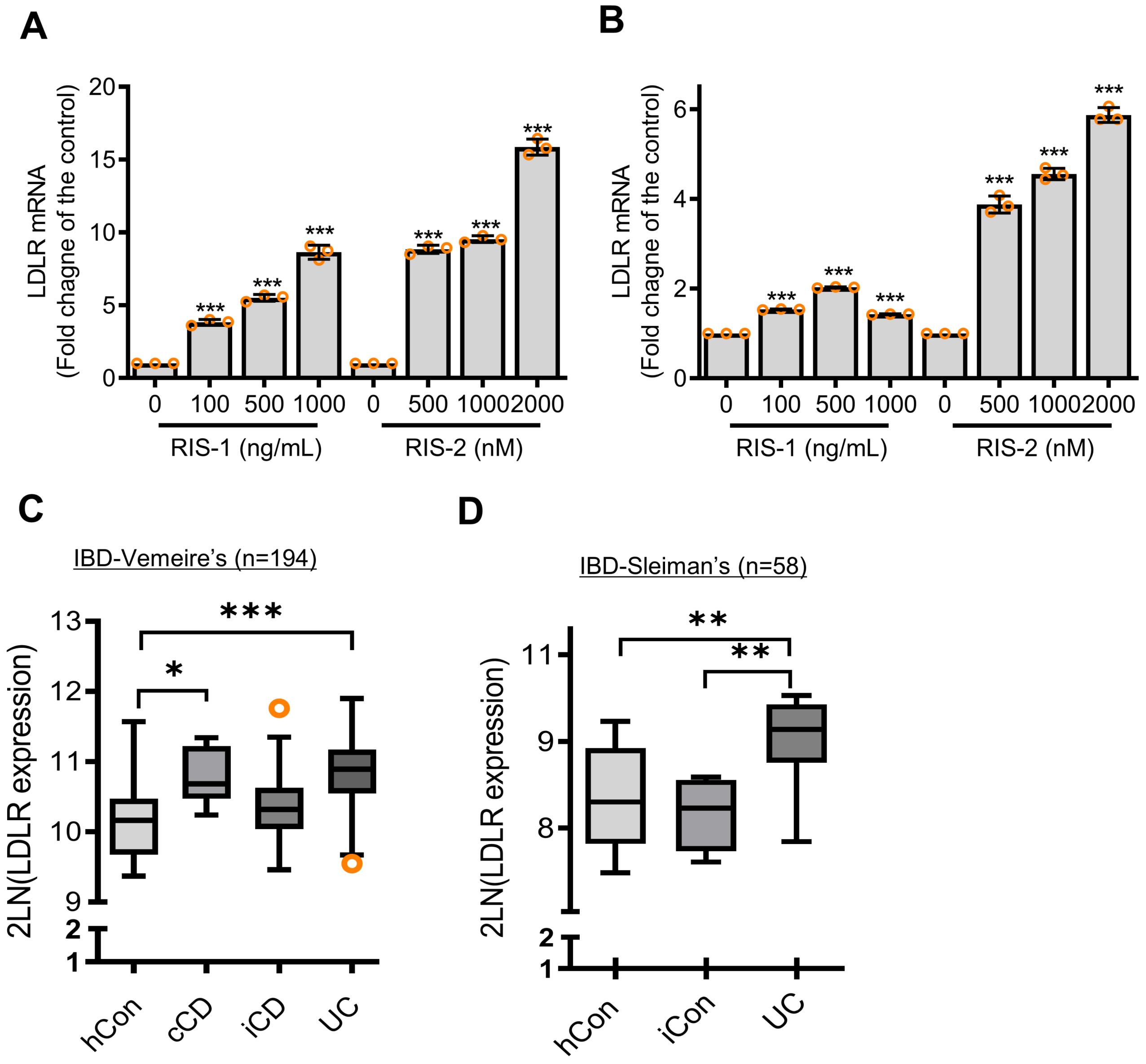

Figure S4. (A-B) HCT-8 (A) or HepG2 (B) cells were treated with vehicle, RIS-1 (1000 ng/mL), or RIS-2 (2 mM) for 2 h. Each mRNA level was measured using reverse transcription-quantitative PCR. Different letters over each bar represent significant differences between groups ($p < 0.05$). "*"$p<0.05$, "**"$p<0.01$, "***"$p<0.001$. (C-D) Intestinal expression of LDLR was compared in patients with different types of IBD datasets (gse75214 (C, *Vemeire*'s, n=194) and gse10616 (D, *Sleiman*'s, n=58)). Asterisks (*) indicate significant differences from the control groups (the healthy control [hCon] or the internal control [iCon]) ($p<0.05$ *, $p<0.01$ **, $p<0.001$ ***). IBD, inflammatory bowel disease; LDL, low-density lipoprotein; LDLR, low-density lipoprotein receptor.

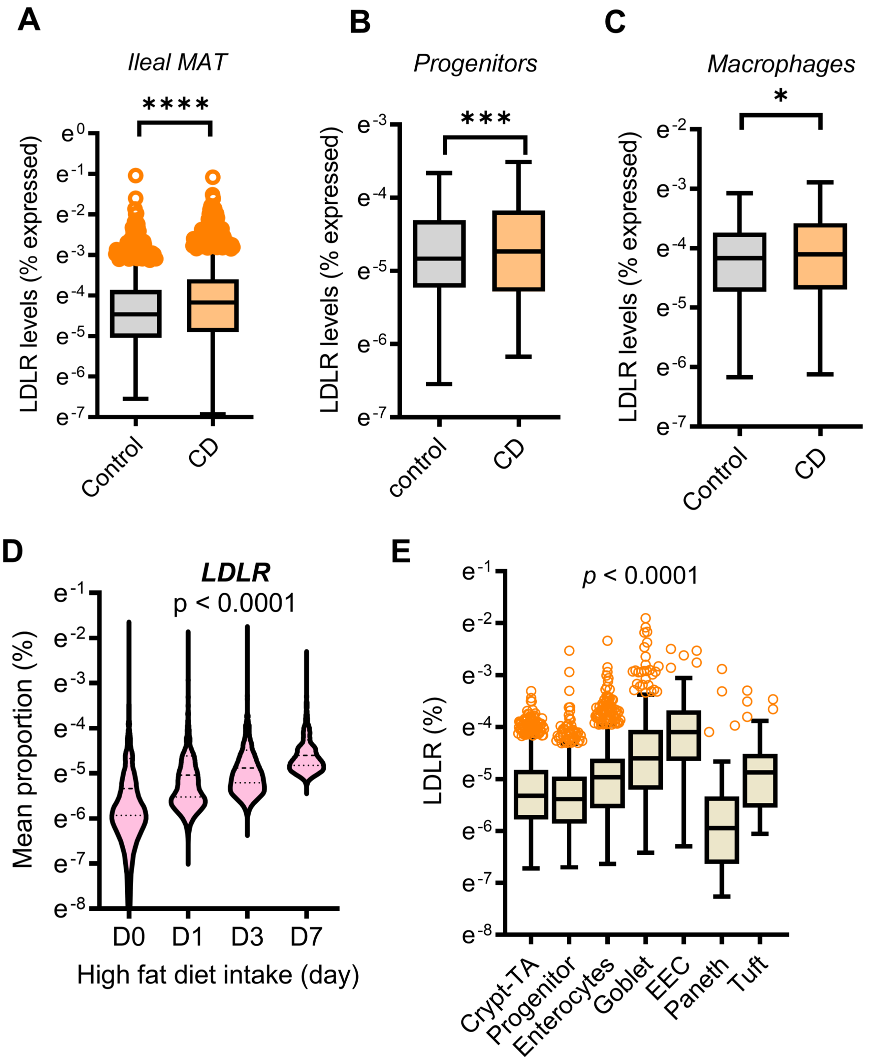

Figure S5. (A-C) Analysis based on scRNA-Seq dataset from patients with CD (gse15677). *LDLR* expression was assessed in ileal mesenteric adipose tissue (MAT, A), progenitor cells (B), and MAT macrophages (C) from patients with CD. Results are shown as a box-and-whisker plot (Tukey), and asterisks (*) indicate significant differences from the low expression group (*p<0.05, *** p<0.001, **** p<0.0001). (D-E) Analysis based on scRNA-Seq dataset from mouse intestine fed with HFD for 7 days (gse199776). Gene expression levels in whole intestinal cells with time (D) or different types of intestinal cells (E). Results are shown as Violin plots and a box-and-whisker plot (Tukey).

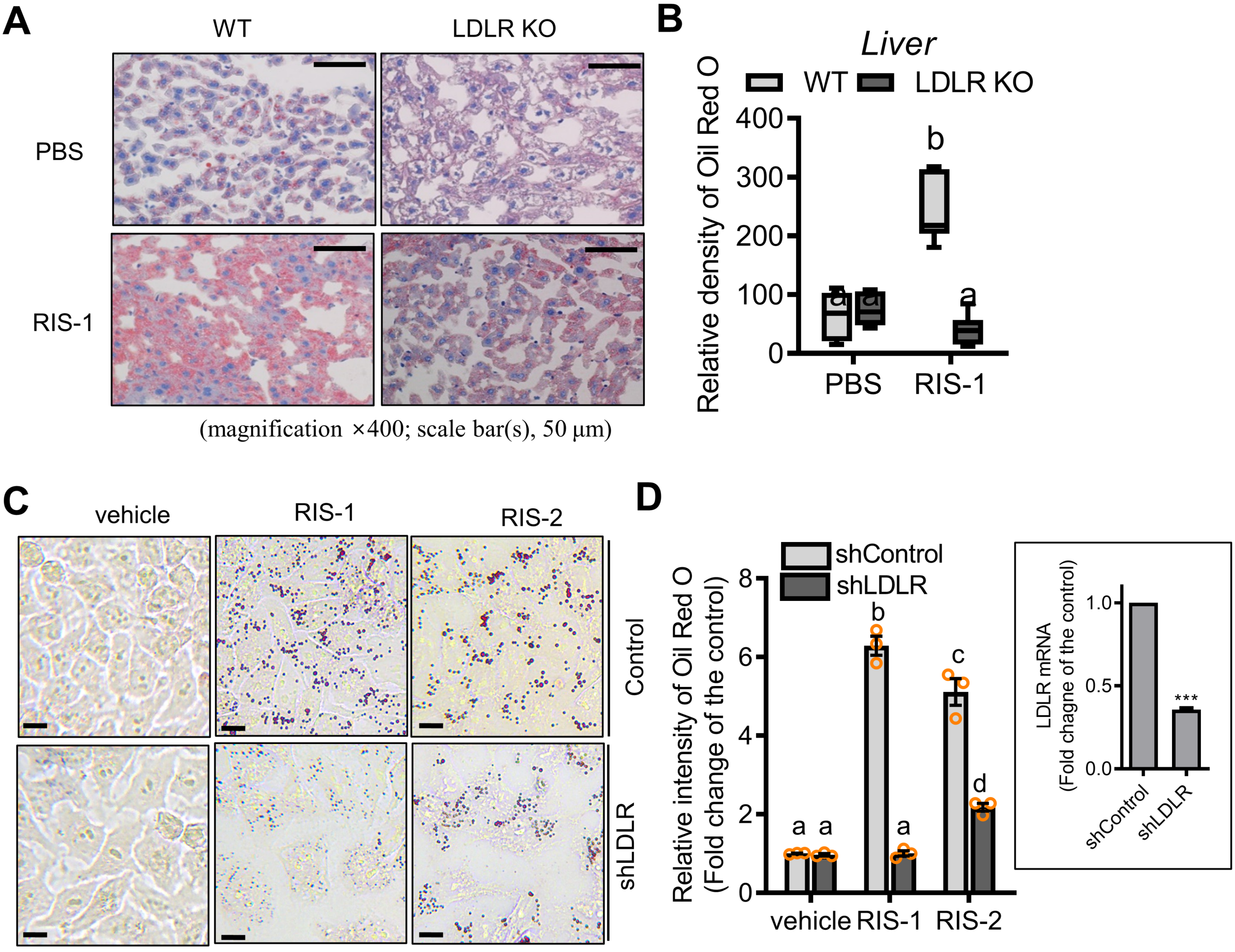

Figure S6. (A-B) Ten-week-old male C57BL/6J wild type or LDLR KO mice were treated with the vehicle or 25 mg/kg RIS-1 for 24 h via oral gavage. Representative Oil Red O staining for the liver. (B) Relative quantitative values of Oil Red O-positive lipid droplets in the liver were using image J software. The microscopy analysis was performed at original magnification x400; scale bar(s), 50 μm. Results are shown as box-and-whisker plots (Turkey) and different letters over each box represent significant differences between groups ($p < 0.05$ using one-way ANOVA with the Newman–Keuls post hoc test). (C-D) HepG2 cells transfected with the negative control vector or shLDLR were treated with the vehicle, 1000 ng/mL RIS-1, or 2 μM RIS-2. Intracellular lipid droplets were stained with Oil Red O and visualized by light microscope. The microscopic analysis was performed at original magnification x200; scale bar(s), 50 μm (left panel). (D) The graph shows relative quantitative values of Oil Red O-positive lipid droplets in cells using image J software. The boxed graph represents the suppression of LDLR mRNA expression by shLDLR (***$p < 0.001$).

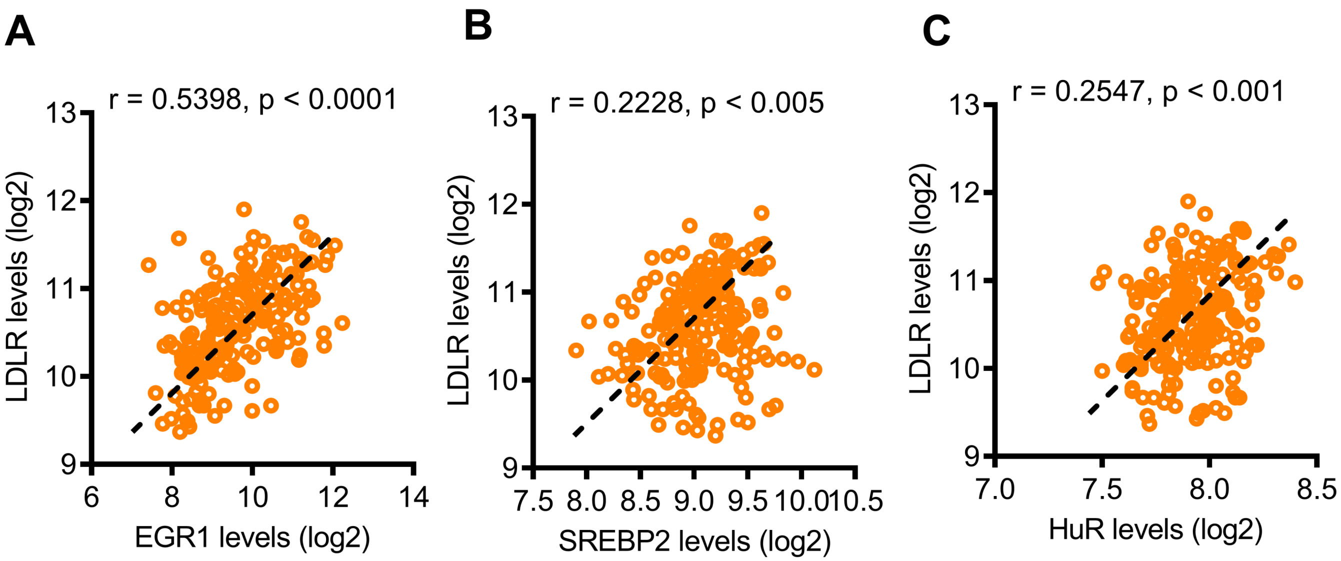

Figure S7. Intestinal expression of *LDLR* was correlated with levels of identified signaling mediators (*EGR1 (A), SREBP2 (B)*, or *HuR (C*) in IBD patients (gse75214 (*Vemeire*'s, n=194)) based on *Pearson* correlation analysis.

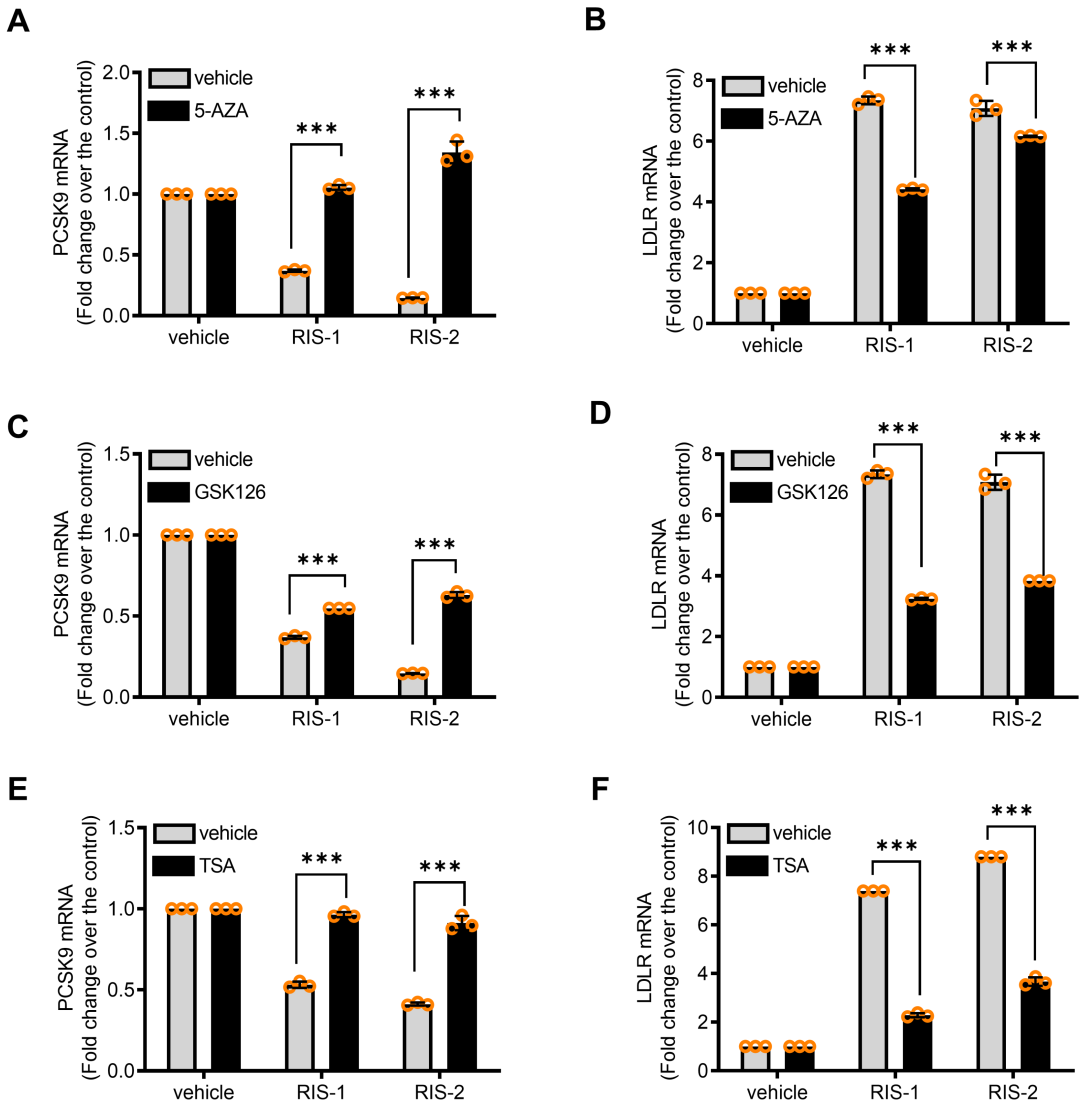

Figure S8. HCT-8 cells were pre-treated with the vehicle or each inhibitor (80 nM TSA (A-B), 4 mM 5-AZA (C-D), or 2 mM GSK126 (E-F)) for 2 h and then exposed to vehicle or RIS-1 (1000 ng/mL), or RIS-2 (2 mM) for 2 h. Each mRNA levels were measured using reverse transcription-quantitative PCR. Asterisks (*) indicate significant differences between groups ($p < 0.05$). "***"$p<0.001$. LDLR, low-density lipoprotein receptor; PCSK9, Proprotein Convertase Subtilisin/Kexin Type 9.
